# TBK1 and IKKε prevent premature cell death by limiting the activity of both RIPK1 and NLRP3 death pathways

**DOI:** 10.1126/sciadv.adq1047

**Published:** 2025-03-07

**Authors:** Fabian A. Fischer, Benjamin Demarco, Felicia Chan Hui Min, Hui Wen Yeap, Dominic De Nardo, Kaiwen W. Chen, Jelena S. Bezbradica

**Affiliations:** ^1^ The Kennedy Institute of Rheumatology, University of Oxford, Oxford, UK.; ^2^ Immunology Translational Research Programme, Department of Microbiology and Immunology, Yong Loo Lin School of Medicine, National University of Singapore, Singapore, Singapore.; ^3^ Department of Biochemistry and Molecular Biology, Monash Biomedicine Discovery Institute, Monash University, Clayton, Victoria, Australia.

## Abstract

The loss of TBK1, or both TBK1 and the related kinase IKKε, results in uncontrolled cell death–driven inflammation. Here, we show that the pathway leading to cell death depends on the nature of the activating signal. Previous models suggest that in steady state, TBK1/IKKε-deficient cells die slowly and spontaneously predominantly by uncontrolled tumor necrosis factor–RIPK1–driven death. However, upon infection of cells that express the NLRP3 inflammasome, (e.g., macrophages), with pathogens that activate this pathway (e.g., *Listeria monocytogenes*), TBK1/IKKε-deficient cells die rapidly, prematurely, and exclusively by enhanced NLRP3-driven pyroptosis. Even infection with the RIPK1-activating pathogen, *Yersinia pseudotuberculosis*, results in enhanced RIPK1–caspase-8 activation and enhanced secondary NLRP3 activation. Mechanistically, TBK1/IKKε control endosomal traffic, and their loss disrupts endosomal homeostasis, thereby signaling cell stress. This results in premature NLRP3 activation even upon sensing “signal 2” alone, without the obligatory “signal 1.” Collectively, TBK1/IKKε emerge as a central brake in limiting death-induced inflammation by both RIPK1 and NLRP3 death-inducing pathways.

## Teaser

TBK1 and IKKe prevent premature cell death by limiting the activity of multiple death pathways in myeloid cells.

## INTRODUCTION

TBK1 (TANK binding kinase 1) and IKKε [inhibitor of nuclear factor κ B (NF-_K_B) kinase subunit ε] are two central kinases in the regulation of several cellular processes and immune pathways including autophagy, metabolism, antiviral immune responses, and cell death pathways. They are two highly related kinases, and the loss of one is often compensated by the other, for instance, in cGAS-STING [cyclic guanosine monophosphate–adenosine monophosphate synthase (cGAS)–stimulator of interferon genes (STING)]–mediated NF-_K_B activation (*1*), TLR (Toll-like receptor)–induced glycolysis (*2*), control of premature cell death by RIPK1 (receptor-interacting serine/threonine kinase 1)–dependent apoptosis or necroptosis (*3*–*5*), or control of NLRP3 (NLR family pyrin domain–containing 3)–driven pyroptosis (*6*). Despite their well-reported function in antiviral defense, the emerging role of TBK1/IKKε in limiting premature cell death appears to be dominant over the other roles of TBK1 and nonredundant in vivo in humans and mice.

Humans expressing loss-of-function (LoF) TBK1 mutations do not suffer from excessive viral infections but do suffer from excessive cell death–driven inflammation in a steady state largely mediated via uncontrolled TNF (tumor necrosis factor)–RIPK1–driven cell death (*7*). In agreement, mice deficient in TBK1 are embryonically lethal because of aberrant tonic TNF- and RIPK1-driven cell death, which can be rescued by crossing mice to either TNF signaling–deficient [TNF/TNF receptor 1 (TNFR1) knockout (KO)] or to RIPK1 kinase activity–deficient mice (*3*, *8*–*11*). However, mice with genetic deletion of both TBK1 and IKKε or with expression of both kinase-dead mutant forms cannot be fully rescued by TNF deficiency and display inflammation caused by combined RIPK1-driven death and excessive IL-1β (interleukin-1β) production by a yet undefined mechanism in myeloid cells (*5*, *12*). Recently, we reported that pharmacological inhibition or small interfering RNA (siRNA) deletion of both TBK1 and IKKε in macrophages sensitizes these cells to NLRP3-driven pyroptosis, in the presence of pathogen- or tissue damage–derived inflammasome-activating ligands, resulting in excessive cell death and IL-1β release. The cytokine release and cell death were specific to NLRP3 but not other inflammasomes and resulted in premature activation of cell death in both mouse and human macrophages (*6*). Together, our work and that of others suggested that TBK1/IKKε may act as a common brake limiting premature activation of multiple cell death pathways in myeloid cells. Our work also opened an important question to be tested here: Under which conditions do cells lacking TBK1/IKKε function die by the RIPK1- versus NLRP3-driven pathway?

NLRP3 is an intracellular receptor protein of the inflammasome family that acts as a sensor for the loss of cellular homeostasis during infections and sterile injury. NLRP3 activation is crucial for resolving infections and instructing adaptive immune responses, yet if dysregulated because of inherited NLRP3-activating mutations or in acquired diseases such as Gouty arthritis or Alzheimer’s disease, it becomes detrimental and results in aberrant inflammatory episodes (*13*). NLRP3 is activated in a two-step process, which requires a priming step (signal 1) for its transcriptional up-regulation and posttranslation modifications (*14*, *15*) and an activation step (signal 2) to assemble the inflammasome complex resulting in ASC speck formation (signaling adapter, apoptosis-associated speck-like protein containing a CARD), caspase-1 activation (effector enzyme), IL-1β and IL-18 secretion, and pyroptotic cell death. While different priming signals regulate NLRP3 transcriptionally via NF-κB signaling (*14*), the activation signals are diverse in their mechanism and are largely classified into signals mediating potassium efflux, reactive oxygen species production, or organelle damage (*16*). More recently, several studies suggested that diverse NLRP3-activating signals 2 all disrupt endosomal–endoplasmic reticulum (ER) contact sites (EECS) and normal vesicle trafficking, resulting in the accumulation and dispersion of intracellular endocytic vesicles. These vesicles also accumulate the negatively charged phospholipid phosphatidyl-4-phosphate (PI4P), and this provides a platform for charge-dependent recruitment of NLRP3 and downstream activation (*17*–*19*). We reported previously that TBK1/IKKε control NLRP3 activation upstream of ASC speck formation, but how precisely they do so remained unclear and was investigated in this study.

Here, we show that acute pharmacological inhibition or full genetic deletion of TBK1/IKKε sensitized macrophages to rapid, premature, and excessive death mediated exclusively by enhanced NLRP3, and not RIPK1 signaling, upon infection with the NLRP3-activating pathogen, *Listeria monocytogenes.* We found the same when macrophages were activated by purified NLRP3 activators, such as the potassium efflux–dependent pore-forming toxin nigericin, the alarmins ATP (adenosine triphosphate) or MSU (monosodium urate), and the potassium efflux–independent TLR7 agonist R837. In addition, even infection of macrophages with the classical RIPK1/caspase-8–activating pathogen, *Yersinia pseudotuberculosis*, sensitized TBK1/IKKε-inhibited cells to enhance RIPK1/caspase-8 activation and death but also enhanced secondary caspase-1 activation. The secondary caspase-1 activation was downstream of RIPK1/caspase 8 signaling (*20*, *21*) (as it was blocked by RIPK1 inhibition) and in TBK1/IKKε-inhibited cells was likely triggered by potassium efflux and NLRP3 activation (as it was also reversed to baseline by NLRP3 deletion). Mechanistically, we found that loss of TBK1 and IKKε lowered the threshold for NLRP3 activation through a cell-intrinsic, transcription-independent process by controlling endosomal homeostasis. The loss of TBK1/IKKε resulted in endosomal TGN46^+^ (trans-Golgi network protein) vesicle accumulation and dispersion, thereby lowering the threshold of NLRP3 activation by both NLRP3-activating and RIPK1-activating pathogens. Disruption of endosomal homeostasis by deletion of another key endosomal regulator Rab5 recapitulated the phenotype of TBK1/IKKε-deficient macrophages, supporting the key role of normal endosomal turnover in preventing premature NLRP3 activation and cell death.

## RESULTS

### TBK1/IKKε limit NLRP3-driven cell death independently of TNF-RIPK1 signaling

**Fig. 1.**
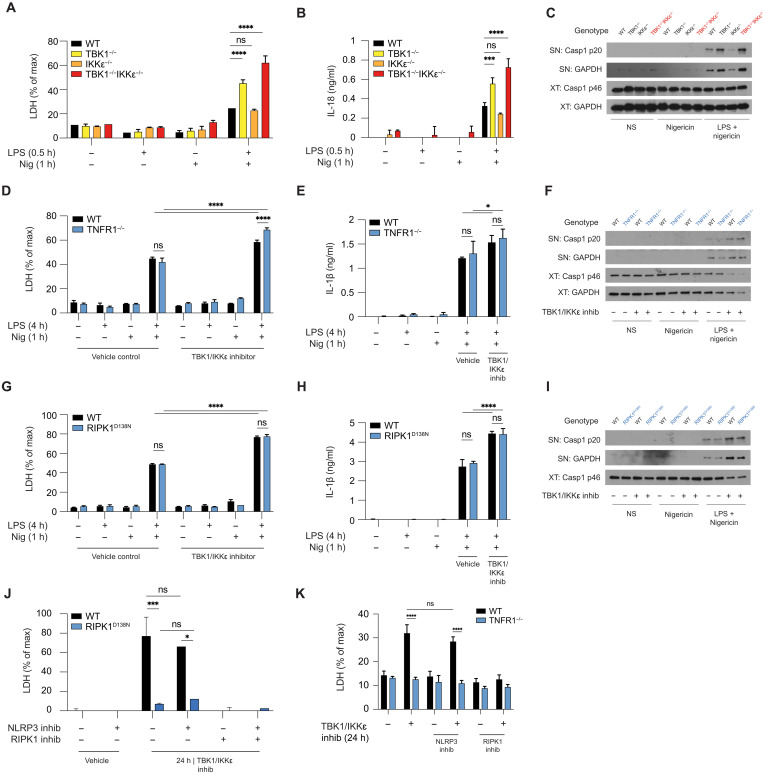
TBK1/IKKε limit NLRP3-driven cell death independently of TNF-RIPK1 signaling. (**A** to **C**) WT, TBK1 KO (Tbk1^−/−^), IKKε KO (Ikkε^−/−^), or dual TBK1 (Tbk1^−/−^Ikkε^−/−^) iBMDMs were primed with LPS (1 μg/ml) for 30 min followed by stimulation with 10 to 15 μM nigericin for 1 hour (h). (**D** to **I**) WT, TNFR1 KO (Tnfr1^−/−^), or RIPK1 kinase–dead (Ripk1^D138N^) BMDMs were primed with LPS (100 ng/ml) for 4 hours followed by stimulation with 7.5 μM nigericin. The cells were treated 30 min before nigericin with 3 μM (target-specific concentration) of the TBK1/IKKε inhibitor MRT68601. (**J** and **K**) WT, RIPK1^D138N^, or TNFR1 KO (Tnfr1^−/−^) BMDMs were incubated for 24 hours with 3 μM MRT68601 in the presence of 10 μM MCC950 (NLRP3 inhib) or 50 μM Nec-1 (RIPK1 inhib). [(A), (D), (G), (J), and (K)] Cell viability was measured using LDH release. [(B), (E), and (H)] Cytokine secretion was measured using enzyme-linked immunosorbent assay. [(C), (F), and (I)] Caspase-1 cleavage in supernatants (SNs) and cell extracts (XTs) was measured using immunoblot. Data are shown as means + SD of duplicates [(A) and (B)] or triplicates [(D), (E), (G), (H), (J), and (K)] from one representative experiment of three independent experiments and one representative immunoblot of three (C) or two shown [(F) and (I)]. ns, not significant.

We had previously shown the role of TBK1/IKKε in limiting NLRP3 activation and cell death using pharmacological inhibition or siRNA deletion of these kinases (*6*). Here, we first confirmed these findings using genetic deletion. TBK1/IKKε dual KO mice are embryonically lethal and cannot be rescued by TNF or TNFR deletion, thereby limiting access to macrophages from these mice (*12*). Thus, to investigate the effect of TBK1/IKKε deletion on NLRP3 activation, we used immortalized bone marrow–derived macrophages (iBMDMs) lacking TBK1, IKKε, or both kinases, previously generated by CRISPR-Cas9–mediated gene targeting (fig. S1A) (*1*). We treated cells (in Fig. 1, A to C) using an acute model of lipopolysaccharide (LPS) priming (30 min) to focus on transcription-independent readouts of inflammasome signaling, i.e., cell death, caspase-1 cleavage, and IL-18 secretion. This allowed us to uncouple previously unknown role of TBK1/IKKε in direct cell death control from the known role of TBK1/IKKε in TLR-induced transcription and up-regulation of proteins such as pro–IL-1β or TNF after chronic LPS priming of 4 hours (fig. S1, B to D). We then stimulated primed macrophages with the bacterial ionophore nigericin. Both *Tbk1*^−/−^ and *Tbk1*^−/−^*Ikk*ε^−/−^ iBMDMs showed increased NLRP3-driven cell death, IL-18 secretion, and caspase-1 cleavage in response to LPS and nigericin compared to wild-type (WT) controls (Fig. 1, A to C), thereby confirming our previous results (*6*).

Immortalized macrophages required higher doses of nigericin than primary macrophages to activate NLRP3, yet regardless of the doses of nigericin used, enhanced NLRP3-driven cell death was conserved in primary and immortalized macrophages when TBK1/IKKε were inhibited (in primary cells) or deleted (in immortalized macrophages) (fig. S1E). These data confirmed that NLRP3-dependent death was elevated in cells when TBK1/IKKε control was lost. In contrast, TBK1/IKKε-deficient cells behaved like WT controls following activation of the AIM2 and NLRC4 inflammasomes in response to transfected calf-thymus DNA or bacterial flagellin, respectively (fig. S1, F and G), supporting the role of TBK1 and IKKε in specific regulation of NLRP3 inflammasome activity. Deletion of IKKε alone only slightly reduced NLRP3 pathway activity (Fig. 1C), reproducing our previous and others’ works (*6*, *22*) and implying an additional role of IKKε in pathway priming. These results further support the dominant role of TBK1 in inhibiting NLRP3 pathway activity, with IKKε deletion only having an effect in combination with TBK1 deletion, where the level of compensation by IKKε may depend on the cell type and relative expression of this kinase. Immune cells, including macrophages, express both TBK1 and IKKε with functional redundancy, while in vivo, many nonmyeloid cells only express TBK1 (*23*, *24*); hence, TBK1 single gene deletion results in lethality and cell death–driven pathology in mice and humans, respectively.

TBK1/IKKε prevent TNF-driven apoptosis/necroptosis by direct inhibitory phosphorylation of RIPK1 (*3*, *4*). To test whether the increase in NLRP3 activity and death in cells lacking TBK1/IKKε is secondary to increased autocrine TNF-RIPK1 signaling, we stimulated BMDMs from TNFR1 KO (*Tnfr1*^−/−^) or RIPK1 kinase–dead (*Ripk1*^D138N/D138N^) mice with LPS for 4 hours (to allow time for TNF secretion) followed by nigericin treatment in the presence or absence of TBK1/IKKε inhibitors. Inhibitors were added 30 min before nigericin and used at target-specific concentrations, established by titration in *Tbk1*^−/−^*Ikk*ε^−/−^ cells (fig. S1I). As expected, *Tnfr1*^−/−^ cells showed no response to TNF stimulation, confirming their genotype (fig. S1H), but both *Tnfr1*^−/−^ and *Ripk1*^D138N/D138N^ were fully responsive to LPS activation (fig. S1, J and K). In the presence of TBK1/IKKε inhibitors, NLRP3-dependent cell death, IL-1β secretion, and caspase-1 cleavage were increased in both WT and *Tnfr1*^−/−^ BMDMs (Fig. 1, D to F). Similarly, NLRP3 responses were equivalent between *Ripk1*^D138N/D138N^ cells and WT controls but were all increased in the presence of TBK1/IKKε inhibitors (Fig. 1, G to I). As expected, *Ripk1*^D138N/D138N^ cells did not die in response to pharmacologically induced necroptosis (fig. S1L). Collectively, these data suggest that TBK1/IKKε regulation of the NLRP3 pathway is independent of TNF-RIPK1.

As reported previously (*4*), we found that prolonged (24 hours) TBK1/IKKε inhibition resulted in spontaneous cell death in the absence of any signal and this death was driven by tonic TNF-RIPK1 signaling (Fig. 1, J and K). These results suggest that in a steady state, TBK1/IKKε-deficient cells die slowly and spontaneously by RIPK1-driven death. However, in contrast, we show that if TBK1/IKKε-deficient cells that also express the NLRP3 inflammasome, such as macrophages, are exposed to signals that can activate this pathway, they die rapidly and exclusively via enhanced NLRP3-driven pyroptosis, independent of TNFR-RIPK1 signaling.

### TBK1/IKKε limit the activity of both RIPK1 and NLRP3 death pathways in macrophages

**Fig. 2.**
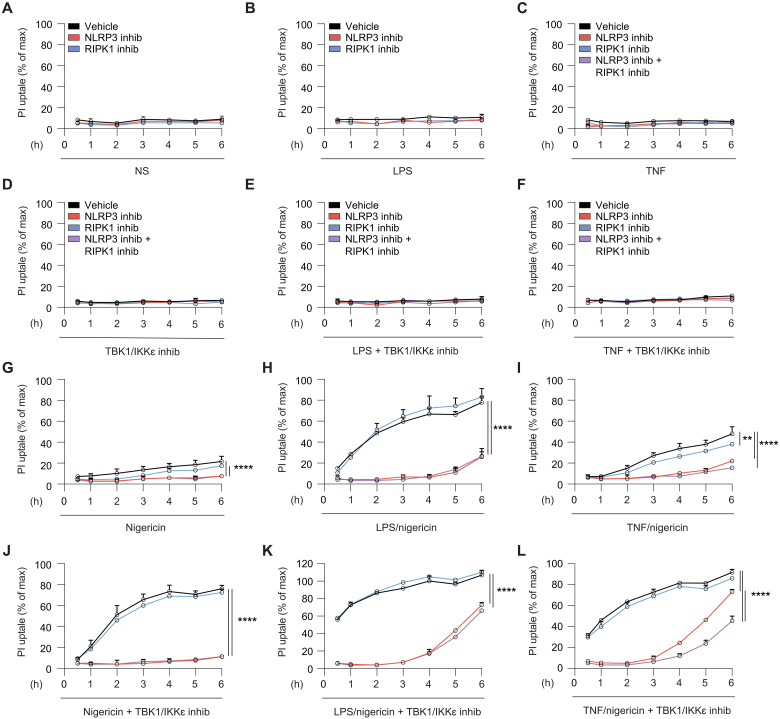
TBK1/IKKε limit the activity of both RIPK1 and NLRP3 death pathways in macrophages. BMDMs were primed with LPS (100 ng/ml) or TNF (100 ng/ml) for 30 min, followed by stimulation with 7.5 μM nigericin for 6 hours. Cells were treated with 3 μM MRT68601, 10 μM MCC950, and 50 μM Nec-1 at the time of priming. After nigericin stimulation, SNs were harvested and cells were lysed for immunoblot analysis. (**A** to **L**) Cell viability was measured every 60 min by measuring PI uptake signals. Data are shown as means + SD of duplicates from one representative of three independent experiments.

Published and our data (Fig. 1, J and K) (*4*) show that in the absence of any external microbial ligands, cells lacking TBK1/IKKε die in 24 hours via unrestrained tonic TNFR/RIPK1 signaling. By contrast, in the presence of microbial activators of the NLRP3 pathway, cells that express NLRP3, such as macrophages, are dead within 60 min, and they die independently of TNFR/RIPK1 pathway (Fig. 1). Data thus suggest that TBK1/IKKε can limit both RIPK1- and NLRP3-driven death in macrophages. To strengthen our conclusion that death of macrophages is exclusively NLRP3 dependent when NLRP3 activators are present, we performed a propidium iodide (PI) uptake assay in a time course experiment where primary BMDMs were primed with LPS (pathogenic signal) or TNF (sterile signal) priming followed by activation with nigericin for 1 to 6 hours. We blocked TBK1/IKKε kinase function using a TBK1/IKKε inhibitor, and we also blocked individual cell death pathways using either NLRP3 and RIPK1 inhibitors or their combination. As expected, unstimulated cells, LPS- or TNF-primed cells, with or without TBK1/IKKε inhibition did not show PI uptake during the 6 hours of stimulation (Fig. 2, A to F), confirming our own (Fig. 1, J and K) and previous data that spontaneous TNF-RIPK1–driven death only occurs after prolonged (>10 hours) TBK1/IKKε inhibition in BMDMs (*4*). Treatment with nigericin alone resulted in a very low-level PI uptake after 6 hours of stimulation (Fig. 2G), while we observed robust cell death following LPS and nigericin treatment or, to a lesser extent, with TNF and nigericin (Fig. 2, H and I). This finding is in line with our previous work that sterile signals such as TNF are generally weaker in priming the NLRP3 pathway when compared with microbial LPS (*25*). In both settings, cell death was NLRP3 dependent but RIPK1 independent (Fig. 2, H and I), where the activity of RIPK1 inhibitor was confirmed in control BMDMs upon pharmacologically induced necroptosis (fig. S2A). All cells pretreated for 30 min with a TBK1/IKKε inhibitor (Fig. 2, J to L) showed rapid and enhanced cell death, even in response to nigericin alone and more so in response to LPS or TNF and nigericin treatment (Fig. 2, J to L). Cell death was exclusively driven by NLRP3, except for a fraction of the cells showing RIPK1-dependent death developing at later time points (after 4 hours) in response to TNF/nigericin stimulation, and a fraction of cells developing MCC950-independent death in LPS/nigericin stimulation when TBK1/IKKε were inhibited (Fig. 2, K and L). We hypothesized that the residual, inhibitor-independent death in these conditions was due to caspase-8 activation at later time points, as BMDMs can initiate an apoptotic caspase-8–driven death when NLRP3 is inhibited (*26*). We detected both cleaved caspase-1 and caspase-8 from cells stimulated with LPS and nigericin after 6 hours, which was greatly enhanced by TBK1/IKKε inhibition. In all conditions, caspase-1 activity remained NLRP3 dependent (fig. S2, B and C). Overall, we conclude that the rapid and increased cell death of macrophages lacking TBK1/IKKε function is fully NLRP3 dependent in the presence of NLRP3-activating signals. Stimulation with TNF and nigericin also triggers rapid NLRP3-driven death but at later time points also involves a partial role for RIPK1-death pathways in the absence of TBK1/IKKε activity.

Mice lacking TBK1 are embryonically lethal and only rescued by TNFR deletion, so previously published in vitro characterization of primary cells from these mice was typically done on a TNF-deficient background (*3*, *8*, *10*). However, our immortalized *Tbk1*^−/−^*Ikk*ε^−/−^ macrophages survived in cell culture even without TNF deletion. Others reported similar observations in *Tbk1*^−/−^*Ikk*ε^−/−^ cell lines [A549 cells (*7*)] and *Tbk1*^−/−^*Ikk*ε^−/−^ fibroblasts (*9*). This could be explained either by low tonic TNF levels produced by these lines in culture or by adaptation of these cell lines to the culture by down-regulation of death pathway–inducing effectors. In support of the latter, we found that our immortalized *Tbk1*^−/−^*Ikk*ε^−/−^ macrophages became selected for cells with lower RIPK3 expression and are more resistant to TNF-induced death when compared to WT (fig. S2, D to F), likely because high RIPK3 expression contributes to lytic cell death downstream of TNFR in TBK1-deficient cells (*3*, *4*) and in some WT settings (*27*, *28*). Previously, others have overexpressed RIPK3 back into *Tbk1*^−/−^*Ikk*ε^−/−^ cell lines (*4*). In this study, we instead used the pharmacological TBK1/IKKε inhibition approach in primary macrophages to explore the role of TNFR, RIPK1, and RIPK3 versus NLRP3 in excessive cell death, where all pathways are endogenously expressed. We then recapitulated the novel, NLRP3-driven biology genetically in immortalized *Tbk1*^−/−^*Ikk*ε^−/−^ macrophages where the NLRP3 pathway is intact (Fig. 1).

### Cell death in response to infection is augmented when TBK and IKKε control is lost

Since the loss of TBK1/IKKε resulted in excessive acute NLRP3–driven death of macrophages in response to isolated pathogenic signals like nigericin (Fig. 2) and spontaneous, slow RIPK1-driven death in response to tonic autocrine TNF signaling (Fig. 1, J and K), we next asked which pathway will be responsible for excessive death when macrophages detect whole pathogens. To address this question, we infected macrophages with a preferentially NLRP3- or a preferentially RIPK1-activating pathogen, *Listeria* and *Yersinia*, respectively, in the presence or absence of TBK1/IKKε inhibition or genetic deletion.

**Fig. 3.**
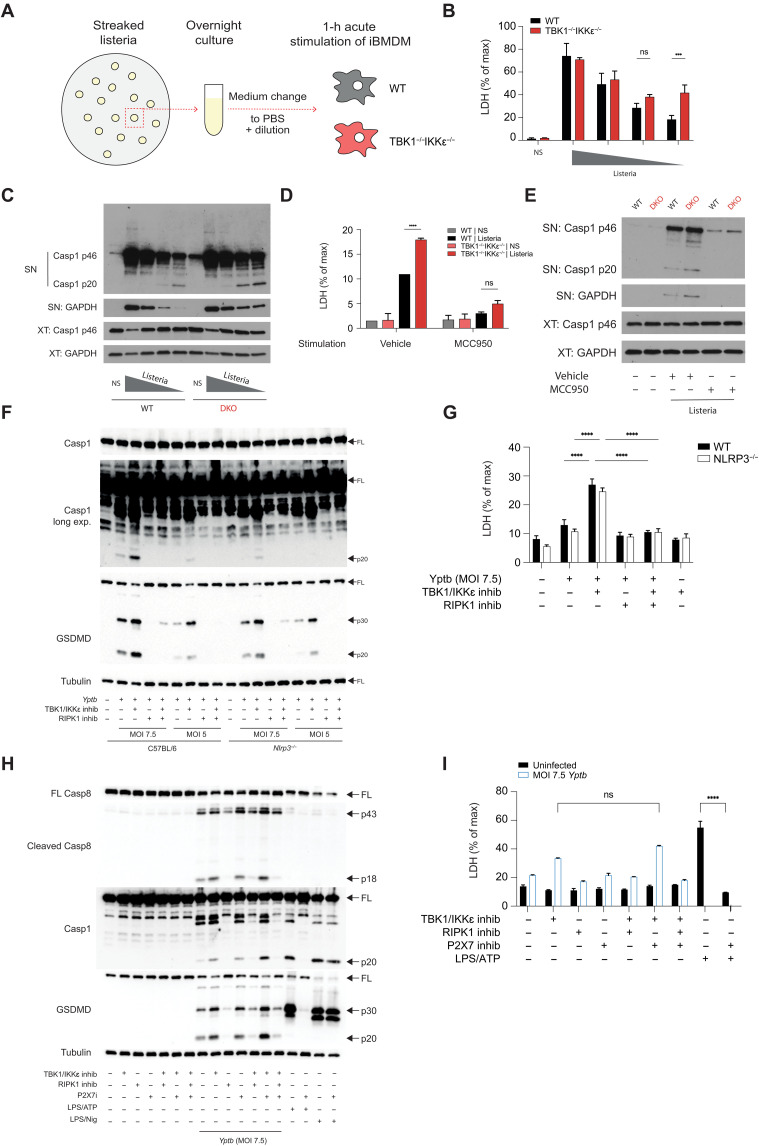
Cell death in response to infections is augmented when TBK and IKKε control is lost. (**A**) Experimental scheme: Overnight cultures of *L.m.* were spun, reconstituted in PBS, and used to acutely infect WT or *Tbk1*^−/−^*Ikk*ε^−/−^ iBMDMs with titrations of *L.m.* for 1 hour, with or without 30-min preincubation with 10 μM MCC950 in (**D** and **E**). [**B** and (D)] Cell viability was measured using LDH release. [**C** and (E)] Caspase-1 cleavage was measured in iBMDM SNs and cell XTs using immunoblotting. (**F** to **I**) Primary BMDMs were infected with *Y. pseudotuberculosis* for 90 min or for 2 hours for the P2X7 inhibitor experiments, and mixed SN and cell XTs were analyzed by immunoblotting. Cell death was measured by LDH release assay. Where indicated, BMDMs were treated with 50 μM Nec-1s or 10 μM MRT67307 or 10 μM P2X7 inhibitor for 30 min before infection. Data are shown as means + SD of infection triplicates (B) or duplicates (D) from one representative experiment of three (B) or two (E) independent experiments and one representative immunoblot in (C) and (E), three experiments in (F) and (G), two of which with MOI 7.5, 5, and 2.5, and one with MOI 5 and 2.5 and two experiments in (H) and (I) with MOI 7.5.

We first infected WT or *Tbk1*^−/−^*Ikk*ε^−/−^ iBMDMs acutely (1 hour) with titrating doses of *L. monocytogenes* for 1 hour. We used an acute 1-hour infection to directly evaluate the TBK1/IKKε effect on cell death responses and separate this direct control of cell death from any later, transcription-dependent roles of TBK1/IKKε in anti-*Listeria* defenses (Fig. 3A) (*29*). In the acute model, the highest doses of *L. monocytogenes* resulted in full and nonspecific lysis of both WT and *Tbk1*^−/−^*Ikk*ε^−/−^ iBMDMs observed by equal LDH (lactate dehydrogenase), GAPDH (glyceraldehyde-3-phosphate dehydrogenase), and full-length caspase-1 release into the supernatant (SN) in the absence of caspase-1 cleavage (Fig. 3, B and C, and fig. S3A). Only at lower, more physiological doses [MOI (multiplicity of infection) 30 to 40], *Listeria* infection activated the NLRP3/caspase-1 pathway and the processed caspase-1 p20 product became evident by immunoblotting. At these doses, when the NLRP3/caspase-1 pathway was active, we observed higher caspase-1 activity and higher cell death in *Tbk1*^−/−^*Ikk*ε^−/−^ macrophages when compared to WT controls (Fig. 3, B and C), and both were fully NLRP3 dependent (Fig. 3, D and E). Thus, these data suggest that when TBK1/IKKε-deficient macrophages (NLRP3-inflammasome competent cells) are exposed to pathogens such as *L. monocytogenes*, they die rapidly via NLRP3-driven pyroptosis, in line with our observations in cells exposed to nigericin alone or LPS and nigericin (Fig. 2).

We next investigated, which cell death pathway would be activated in macrophages infected with the preferentially RIPK1-activating pathogen, *Y. pseudotuberculosis* (*Yptb*), in the context of TBK1/IKKε inhibition. Normally, *Yptb* infection induces RIPK1/caspase-8–driven cell death in BMDMs, which is inflammasome and ASC independent (*30*, *31*). We found that infection of macrophages with the classical RIPK1/caspase-8–activating pathogen, *Yptb*, sensitized TBK1/IKKε-inhibited cells to enhance RIPK1/caspase-8 activation and death (Fig. 3, F and G), likely due to the reported direct inhibitory phosphorylation of RIPK1 by TBK1 (*3*, *4*), but also to enhance secondary caspase-1 activation. The secondary caspase-1 activation was downstream of enhanced RIPK1/caspase-8 signaling (*20*, *21*), in agreement with a recent report (*21*). In our system as well, caspase-1 activation was blocked by RIPK1 inhibition, and in TBK1/IKKε-inhibited cells, it was likely triggered by downstream potassium efflux and NLRP3 activation, as it was also reversed to baseline by NLRP3 deletion (Fig. 3, F and G). Residual caspase-1 activity remained detectable at higher MOI even in NLRP3-deficient BMDMs, which is in agreement with previous reports of some level of direct, NLRP3-independent, caspase-1 activation downstream of the RIPK1/caspase-8 complex during *Ytpb* infection (*21*, *30*). Collectively, these results show that TBK1 and IKKε prevent premature cell death and inflammatory signaling by limiting the activity of both NLRP3 and RIPK1 death pathways in response to NLRP3- and RIPK1-activating pathogens, respectively. Unexpectedly, if macrophages infected with the RIPK1-activating pathogen *Yptb*, also lack TBK1/IKKε activity, this results in enhanced secondary NLRP3 activation, downstream of enhanced RIPK1/caspase-8 signaling.

We next addressed whether secondary, enhanced NLRP3 inflammasome activation occurred in the same/infected cell or the bystander cells because of a lower threshold of activation and increased sensitivity to NLRP3 signals 2 (including ATP) due to the lack of TBK1/IKKε. Thus, we infected WT primary macrophages with *Yptb* in the absence or presence of TBK1/IKKε inhibitors, and we blocked autocrine and paracrine ATP signaling by treating macrophages with the P2X7 inhibitor AZ10606120. As expected, we detected increased RIPK1/caspase-8 activity and increased caspase-1 activity in TBK1/IKKε-inhibited cells. While increased caspase-1 activity and cell death were blocked by the RIPK1 inhibitor Nec1 (suggesting that cell death occurs downstream of RIPK1/caspase-8), they were not blocked by P2X7R inhibition (Fig. 3, H and I). These data therefore suggest that even if caspase-1 activation is triggered in bystander cells, this is not mediated by autocrine or paracrine ATP signaling.

To address the role of other soluble factors in possible bystander cell activation, we next performed an SN transfer experiment from *Yptb*-infected WT cells or Ninjurin-1 lysis–deficient mutants (*Ninj1^K45Q^*). Lytic SN from WT cells, but not NINJ1 mutant cells, induced a low level of caspase-1 activation in noninfected cells, but this was not further increased when TBK1/IKKε were inhibited in these noninfected cells (fig. S3, B to D). Collectively, we conclude that in cells lacking TBK1/IKKε activity, the enhanced RIPK1/caspase-8 activation, the enhanced caspase-1 activation, and cell death likely occur intrinsically within the infected cell, and if caspase-1 is activated in bystander cells, it is not mediated by soluble factors released during cell death. However, at this stage, we cannot rule out a potential role of direct cell contact or short-lived metabolites in bystander cell caspase-1 activation during infection.

### Deletion of TBK1/IKKε allows for priming-independent NLRP3 activation

**Fig. 4.**
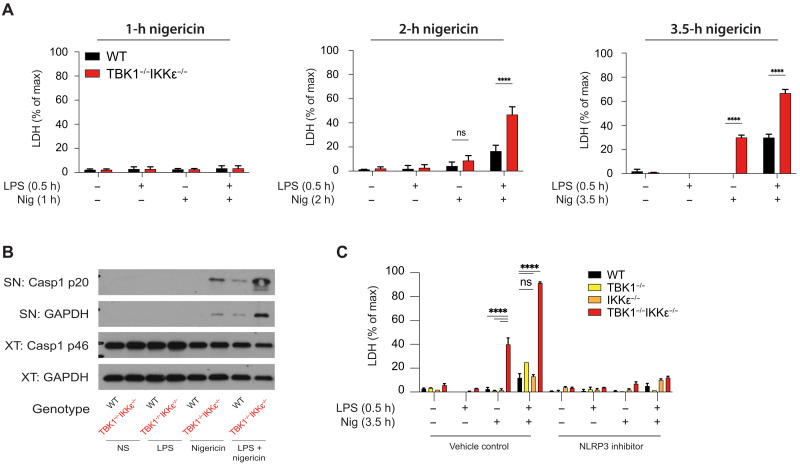
Deletion of TBK1/IKKε allows for priming-independent NLRP3 activation. (**A** to **C**) WT or Tbk1^−/−^Ikkε^−/−^ iBMDMs were primed with LPS (1 μg/ml) for 30 min followed by stimulation with 10 to 15 μM nigericin for 3.5 hours in the presence of 10 μM of the NLRP3 inhibitor MCC950 in (C). [(A) to (C)] Cell viability was measured using LDH release. (B) Caspase-1 cleavage was measured in SN and lysates using immunoblotting. Data are shown as means + SD of triplicates (A) or duplicates (C) from one representative experiment of three independent experiments and one representative immunoblot in (B).

During this study and in our previous work (*6*), we observed that inhibition of TBK1 and IKKε sensitized murine and human macrophages to premature NLRP3 activation and cell death, even in the absence of an obligatory priming signal (Fig. 2J and fig. S4, A to G). To test whether genetic deletion of TBK1 and IKKε recapitulates the phenotype revealed by inhibitors, we stimulated WT and *Tbk1*^−/−^*Ikk*ε^−/−^ iBMDMs with LPS and nigericin or nigericin alone and measured LDH for over 1 to 3.5 hours to account for potential differences in cell death kinetics in iBMDMs. As expected, LPS and nigericin stimulation led to increased LDH release after 2 hours from *Tbk1*^−/−^*Ikk*ε^−/−^ iBMDMs compared to their WT controls. From 2 hours onward, nigericin treatment alone, induced a pyroptotic morphology, followed by cell death, LDH release, and caspase-1 cleavage, in non-primed *Tbk1*^−/−^*Ikk*ε^−/−^ iBMDMs but not in WT iBMDMs controls (Fig. 4, A and B). The cell death observed was entirely NLRP3 dependent (Fig. 4C and fig. S4D) and was also recapitulated with other potassium efflux–dependent (nigericin, ATP, and MSU) and –independent (R837) inflammasome activators (signal 2) (fig. S4, A to G). These results demonstrate that the deletion of TBK1/IKKε substantially lowers the threshold of NLRP3 pathway activation to signal 2 alone, making the requirement for a typical priming step redundant.

### TBK1/IKKε regulate NLRP3 activation via a cell-intrinsic mechanism

TBK1 and IKKε inhibit RIPK1 activation by direct phosphorylation at multiple sites (*3*, *4*), yet the mechanism of how they regulate NLRP3 activation remains incompletely understood. We previously identified TBK1/IKKε in a search for kinases that oppose the activity of PP2A (protein phosphatase 2), the reported phosphatase that dephosphorylates NLRP3 at the serine residue 3, which is a critical requirement for pathway activation. While we confirmed a key role for serine 3 dephosphorylation in NLRP3 activation (*6*), both kinases and the phosphatase maintained control over the pathway, even when serine 3 was mutated to a nonphosphorylated mutant (*6*), suggesing that there must be an aditional place of their action in the NLRP3 pathway. We have also shown that TBK1/IKKε control the pathway upstream of ASC speck formation, so we next explored this mechanism further.

**Fig. 5.**
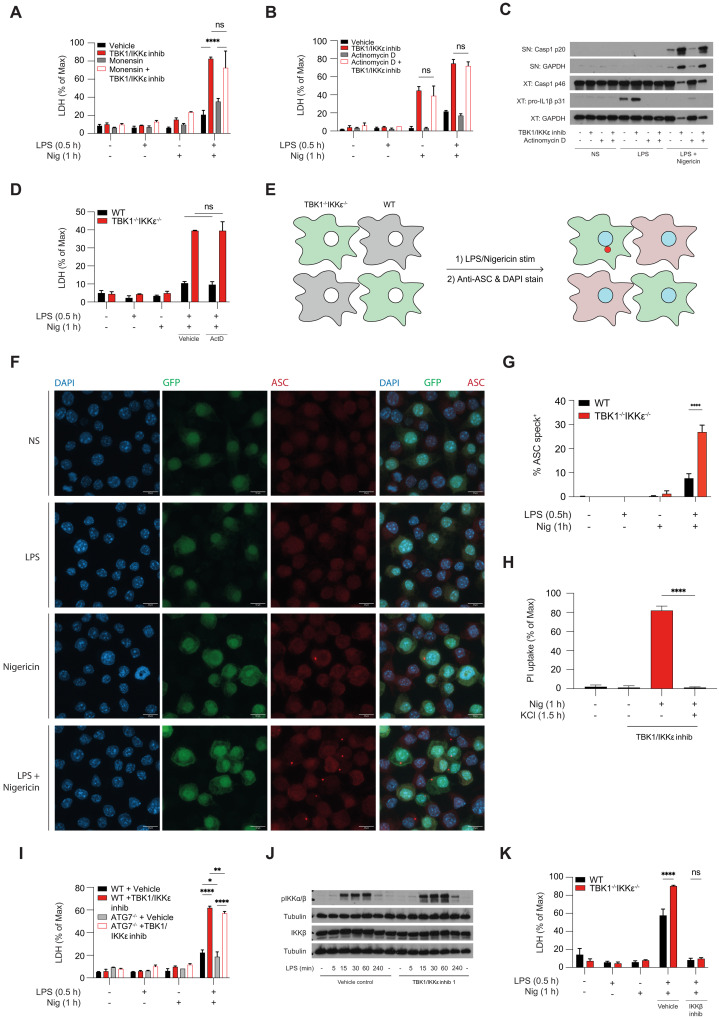
TBK1/IKKε regulate NLRP3 activation via a cell-intrinsic mechanism. (**A**, **B**, **C**, **H**, and **I**) BMDMs were primed with LPS (100 ng/ml) for 30 min followed by stimulation with 7.5 μM nigericin for 1 hour. (**J**) BMDMs were primed with LPS (100 ng/ml) in a time course from 5 min to 4 hours. [(D) and **K**] WT or *Tbk1*^−/−^*Ikk*ε^−/−^ iBMDMs were primed with LPS (1 μg/ml) for 30 min followed by stimulation with 10 to 15 μM nigericin for 1 hour. The cells were pretreated with 3 μM MRT68601 (TBK1/IKKε inhib) for 30 min before LPS treatment, actinomycin D (5 μg/ml) or 2 μM monensin for 45 min before LPS treatment, 5 μM BI605906 (IKKβ inhib) for 1 hour before LPS treatment. Cell viability was measured using LDH release [(A), (B), (D), (I), and (K)] or PI uptake (H). [(C) and (J)] Caspase-1 cleavage was measured in SNs and lysates using immunoblotting. [(E) to (G)] WT or *Tbk1*^−/−^*Ikk*ε^−/−^ iBMDMs were cocultured in the same dish for 24 hours before being primed with LPS (1 μg/ml) for 30 min followed by stimulation with 15 μM nigericin for 1 hour in the presence of 10 μM of the caspase-1 inhibitor VX-765 to prevent lytic cell death. ASC specks were visualized by confocal imaging using an anti-ASC antibody and 4′,6-diamidino-2-phenylindole (DAPI) stain or WT (GFP^−^) and *Tbk1*^−/−^*Ikk*ε^−/−^ (GFP^+^) iBMDMs. Data are shown as collapsed Z-stacks of representative images and quantification showing % cells forming ASC specks from multiple fields of view from three independent experiments is on the right. Data are shown as means + SD of triplicates [(A), (B), (H), and (I)] or duplicates [(D) and (K)] from one representative experiment of two [(A), (I), and (K)] or three [(B), (D), and (H)] independent experiments, as means + SEM from three independent experiments (G) and one representative immunoblot in (C) and (J).

We first examined whether deletion of TBK1/IKKε resulted in the secretion of proteins (e.g., TNF or others) that would act in an autocrine or paracrine way to enhance NLRP3 activity and death. To this end, we pretreated BMDMs with the protein secretion inhibitor monensin and acutely stimulated them with LPS and nigericin. As expected, monensin potently blocked secretion of TNF (fig. S5A) but did not prevent the increase in NLRP3-driven death in cells lacking TBK1/IKKε (Fig. 5A). We obtained similar results in brefeldin-treated cells; however, brefeldin treatment itself slightly reduced NLRP3 activation, even in control BMDMs and iBMDMs (fig. S5, B to F), due to its known influence on endosomal trafficking, the known site of NLRP3 activation (*32*).

Next, we addressed whether TBK1/IKKε control of NLRP3-driven death was dependent on transcription. We, therefore, pretreated BMDMs with the transcriptional inhibitor, actinomycin D, before acute LPS and nigericin stimulation. As expected, actinomycin D potently blocked pro–IL-1β induction but did not affect enhanced NLRP3 activity and death in BMDMs treated with TBK1/IKKε inhibitors (Fig. 5, B and C) nor in *Tbk1*^−/−^*Ikk*ε^−/−^ iBMDMs (Fig. 5D). This strongly suggests that TBK1/IKKε regulate NLRP3 activation via a transcription-independent mechanism.

To rule out any other secreted, soluble factors (e.g. metabolites, lipids, pH, etc.), we set up an extended overnight coculture of WT and *Tbk1*^−/−^*Ikk*ε^−/−^ iBMDMs before activating the NLRP3 inflammasome, with the idea that if *Tbk1*^−/−^*Ikk*ε^−/−^ produce any secreted factors or metabolites that “prime” the NLRP3 pathway, similar NLRP3 responses (as measured by ASC speck formation) should be observed in WT and *Tbk1*^−/−^*Ikk*ε^−/−^ after prolonged coculture. This was not the case, and the frequency of cells forming ASC specks in response to LPS/nigericin was always higher in the *Tbk1*^−/−^*Ikk*ε^−/−^ population [endogenously marked with green fluorescent protein (GFP) expression] than in WT cells cocultured in the same dish (Fig. 5, E to G). This was also true, to some extent with nigericin alone (fig. S5, G and H), albeit specks were less frequent, because of the expected slower activation (2 hours to form ASC specks after signal 2 alone versus 1 hours after signal 1 + 2) and even in the presence of caspase-1 inhibitors, cell death began to interfere with the speck number readout. Last, we recapitulated these findings by transferring SNs between cultured WT and *Tbk1*^−/−^*Ikk*ε^−/−^ iBMDMs (fig. S5, J and K) before acute LPS and nigericin stimulation. Again, we observed no change in the phenotype, supporting a model that cells lacking TBK1/IKKε have a lower threshold of NLRP3 pathway activation and that this happens via a cell-intrinsic mechanism.

We previously mapped the site of TBK1/IKKε action upstream of ASC oligomerization (*6*). To test whether the death of cells lacking TBK1/IKKε in response to signal 2 alone followed the canonical route of NLRP3 activation, we exposed WT or TBK1/IKKε-inhibited cells to either potassium-low or -high medium (5 or 50 mM potassium chloride respectively, KCl) before stimulating them with nigericin alone. The cell death induced by nigericin was fully blocked in the high-potassium medium (Fig. 5H), demonstrating that potassium efflux remains a conserved requirement for the premature NLRP3-driven death in cells lacking TBK1/IKKε.

TBK1 and IKKε activation induces autophagic flux by activating autophagy receptors (*33*–*35*), and autophagy has been previously proposed as a mechanism of NLRP3 removal (*36*), as well as removal of damaged organelles (*37*). To test whether autophagy impairment in cells lacking TBK1/IKKε activity may be responsible for elevated NLRP3 responses, we stimulated BMDMs from autophagy-deficient *Atg7*^−/−^ (autophagy-related 7) mice or WT controls with LPS and nigericin in the presence of TBK1/IKKε inhibition. As expected, autophagy flux was blocked in these cells, and bafilomycin A treatment did not induce the accumulation of LC3-II typically seen in WT controls (fig. S5G), yet ATG7 deletion did not enhance NLRP3 activity and death in response to LPS and nigericin in WT cells, nor did it affect inflammasome activation and cell death response of in TBK1/IKKε-inhibited cells (Fig. 5I and fig. S5, H to J). Hence, impaired autophagy is unlikely to explain the rapid and enhanced death of cells lacking TBK1/IKKε activity, at least under the conditions examined.

Last, recent work demonstrated that NLRP3 recruitment to TGN38^+^ (in mice) or TGN46^+^ (in human) vesicles is essential for its activation (*17*, *18*). This recruitment depends on the canonical IKK kinase, IKKβ, by an as yet unknown mechanism (*38*, *39*). TBK1 and IKKε are known negative regulators of IKKβ signaling (*40*), and, in line with this, we found enhanced IKKβ activation by LPS in cells lacking TBK1/IKKε activity (Fig. 5J). To test whether the increase in NLRP3 activity and cell death in *Tbk1*^−/−^*Ikk*ε^−/−^ iBMDMs is IKKβ dependent, we stimulated these iBMDMs in the presence of an IKKβ inhibitor. As reported, IKKβ inhibition completely prevented NLRP3 activity regardless of genotype (Fig. 5K). However, this also prevented us from further investigating the key importance of IKKβ during NLRP3 activation. Overall, these data show that TBK1/IKKε regulate NLRP3 by a cell-intrinsic mechanism, downstream of potassium efflux, upstream of ASC speck formation, and likely upstream of IKKβ, but independent of new transcription and autophagy.

### Loss of TBK1/IKKε disrupts the endosomal homeostasis and lowers the threshold for NLRP3 activation

**Fig. 6.**
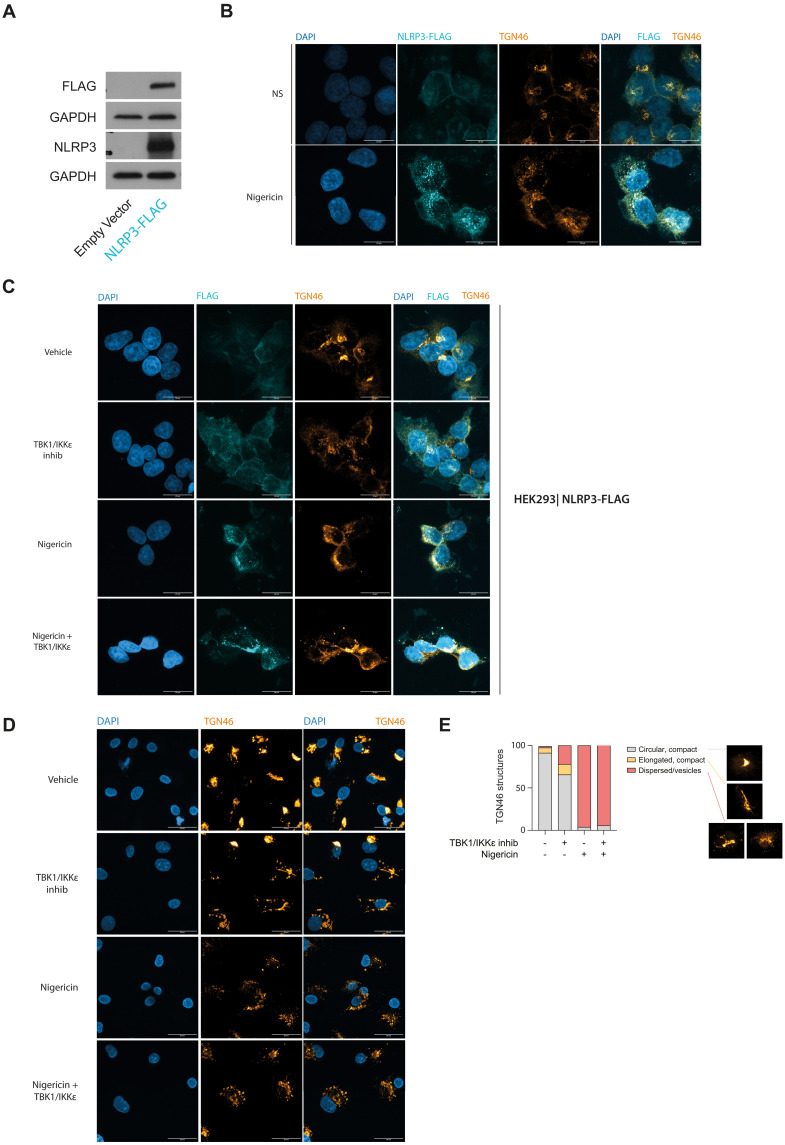
Loss of TBK1/IKKε disrupts the endosomal homeostasis and lowers the threshold for NLRP3 activation. (**A**) HEK293T cells expressing NLRP3-FLAG were generated using lentiviral transduction. (**B** and **C**) HEK293T-NLRP3-FLAG cells pretreated with 3 μM MRT68601 (TBK1/IKKε inhib) were stimulated with 10 μM nigericin for 1 hour. The cells were then fixed and immuno-labeled for TGN46 and FLAG. (**D**) HMDMs were pretreated with 3 μM MRT68601 and 10 μM of the caspase-1 inhibitor VX-765 to prevent lytic cell death followed by stimulation with 7.5 μM nigericin for 30 min. The cells were subsequently fixed and immuno-labeled for TGN46. Data are shown as collapsed Z-stacks of representative images from multiple fields of view from two [(B) and (C)] or three independent experiments for (D). (**E**) Quantification of images from three experiments showing the fraction of cells forming compact, versus elongated versus fully dispersed TGN46 endosomal network (representative shapes shown on the right).

IKKβ is required for the recruitment of NLRP3 to negatively charged lipid PI4P present on TGN46^+^ endocytic membranes (*18*, *39*). To address whether TBK1/IKKε inhibition would result in enhanced NLRP3 recruitment to TGN46^+^ structures, we generated a stable human embryonic kidney (HEK) 293T cell line expressing NLRP3 with a C-terminal FLAG tag (Fig. 6A) and immuno-stained for FLAG and TGN46. As expected, in the absence of an NLRP3 activator, TGN46 was localized into compact, perinuclear structures, while NLRP3-FLAG was diffusely located within the cell. In contrast, following nigericin stimulation, TGN46^+^ vesicles became dispersed and colocalized with NLRP3 (Fig. 6B) in line with previous reports (*18*, *39*).

Next, we pretreated NLRP3-FLAG–expressing cells with a TBK1/IKKε inhibitor before nigericin stimulation and assessed whether NLRP3 redistribution to TGN46^+^ vesicles was affected. Unexpectedly, even the TBK1/IKKε inhibitor alone (i.e., before nigericin stimulation) resulted in the appearance of a partially dispersed TGN46^+^ vesicular network (Fig. 6C). NLRP3 was not recruited to this network until the cells were subsequently treated with nigericin (Fig. 6C). This observation is in line with a previous report that suggests that nigericin is required to expose PI4P on TGN46^+^ vesicles and allow charge-based NLRP3 recruitment. NLRP3-FLAG staining at the network was specific as it was not seen in control HEK293T cells, not expressing NLRP3-FLAG (fig. S6). Next, we assessed whether primary human CD14^+^ monocyte-derived macrophages (HMDMs) would display a similar dispersion of TGN46^+^ vesicles after TBK1/IKKε inhibition explaining our observation that HMDMs treated with TBK1/IKKε inhibitors for 30 min have such a low threshold for NLRP3 activation even in response to nigericin alone (*6*). We found that control, unstimulated HMDMs showed expected compact TGN46^+^ perinuclear structures (Fig. 6D) comparable to HEK293T cells (Fig. 6B). However, similar to HEK293T cells, upon TBK1/IKKε inhibition, TGN46^+^ structures appeared more disperse (Fig. 6, D and E). From these data, we conclude that loss of TBK1/IKKε results in abnormal dispersion of endocytic vesicles, creating a preformed platform for rapid NLRP3 activation, independent of a priming signal.

### Loss of endosomal homeostasis by Rab5 deletion also lowers the threshold for NLRP3 activation

**Fig. 7.**
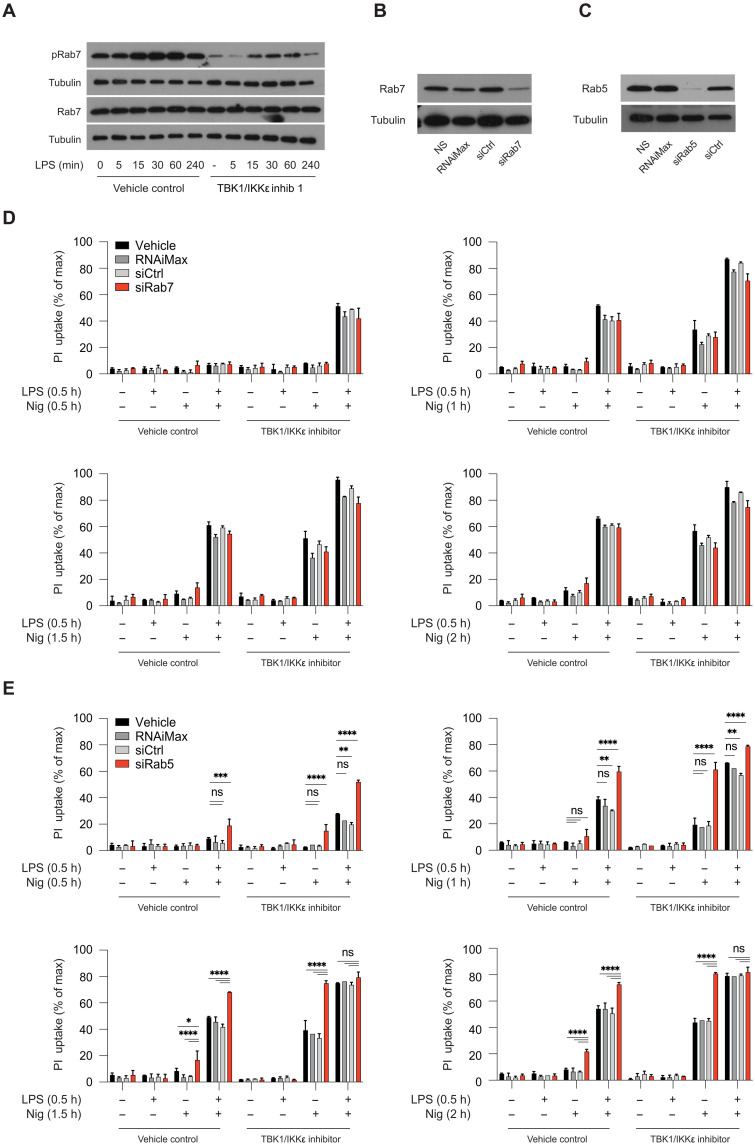
Loss of endosomal homeostasis by Rab5 deletion also lowers the threshold for NLRP3 activation. (**A**) HMDMs were primed with LPS (100 ng/ml) in a time course and pretreated with 3 μM MRT68601 (TBK1/IKKε inhib) for 30 min before LPS treatment. Rab7 activation was assessed by immunoblot for phospho-serine72 levels compared to total Rab7. (**B** to **E**) BMDMs were subjected to siRNA-mediated knockdown of Rab7 [(B) and (D)] or Rab5 [(C) and (E)] for 2 days before replating and stimulation on the third day. BMDMs were primed with LPS (100 ng/ml) and treated with 3 μM MRT68601 for 30 min followed by stimulation with 7.5 μM nigericin for 2 hours. [(B) and (C)] Knockdown efficiency was confirmed using immunoblotting. [(D) and (E)] Cell viability was measured using PI uptake every 30 min over 2 hours. Data are shown as means + SD of duplicates from one representative experiment of three [(D) and (E)] independent experiments and one representative immunoblot for (A) to (C).

The TGN46^+^ vesicular structures forming in response to NLRP3 activators were recently shown to be of endosomal origin caused by disruption of EECS (*18*). Endosomal homeostasis is a process highly dependent on proteins of the family of RAS-related small GTPases (guanosine triphosphates), such as Rab5 and Rab7, required for early and late endosomal function and maturation, respectively (*41*). We therefore tested whether Rab proteins are regulated by TBK1/IKKε and/or whether their deletion would recapitulate the lower threshold of NLRP3 activation and death. Rab7 has been reported to be directly phosphorylated by TBK1 and IKKε in cell lines (*42*, *43*), so we first tested Rab7 phosphorylation in HMDMs after LPS stimulation in the absence or presence of a TBK1/IKKε inhibitor. LPS treatment increased Rab7 phosphorylation, which was strongly reduced in the presence of TBK1/IKKε inhibition (Fig. 7A), suggesting that in macrophages, Rab7 is under the control of TBK1/IKKε. Next, we reduced Rab7 expression by siRNA-mediated knockdown in BMDMs (Fig. 7B) and, after 72 hours, tested NLRP3-driven death using PI uptake, following acute LPS and nigericin or nigericin alone stimulation in the absence or presence of a TBK1/IKKε inhibitor. No notable difference in PI uptake or caspase-1 cleavage was observed between vehicle and Rab7-depleted cells at any time point (Fig. 7D), although low-level caspase-1 cleavage was observed in Rab7-depleted cells with nigericin alone 2 hours poststimulation (fig. S7A). TBK1/IKKε inhibitor treatment increased NLRP3 activation in all conditions (Fig. 7D). Thus, deletion of Rab7, the regulator of late endosomes does not lower the threshold of NLRP3 activation that we have seen with TBK1/IKKε deletion, suggesting that in macrophages, loss of TBK1/IKKε signaling likely arrests the endosomal network traffic a step upstream of Rab7 vesicles.

Early endosomal vesicles originating from disrupted EECS are Rab5^+^ and TBK1 has recently been described to colocalize with and influence the number of early endosomes (*44*), so we next depleted Rab5 using siRNA-mediated knockdown in BMDMs (Fig. 7C). Unexpectedly, this fully recapitulated the premature death phenotype seen with TBK1/IKKε deletion with increased cell death in response to LPS and nigericin at all time points and in response to nigericin alone at later time points. Cell death was again further increased by TBK1/IKKε inhibition (Fig. 7E and fig. S7B). We concluded from these data that any disruption of early endosomal homeostasis, either through loss of TBK1/IKKε or by Rab5 deletion, signals cell stress and lowers the threshold for NLRP3 activation resulting in rapid NLPR3 activation and cell death. It will be interesting to test in the future how TBK1 and IKKε regulate early endosomal homeostasis and whether Rab5 vesicles are directly controlled by TBK1/IKKε in the NLRP3 pathway.

## DISCUSSION

TBK1 and IKKε play a central role in activating proinflammatory cytokine secretion, antiviral immunity, transcriptional responses, and autophagy, all essential to counteract infection and tissue damage and for cells to return to homeostasis. In cells expressing functional TBK1 and IKKε, cell death is blocked so that the cell can execute immune defense functions. However, we propose that loss of TBK1/IKKε function, either due to genetic mutations or during pathogen evasion, acts as an alarm signal to the cell and initiates the removal of the compromised cells by activation of programmed cell death pathways.

So far, most research has focused on understanding the regulation of RIPK1-dependent apoptosis and necroptosis by TBK1/IKKε via direct phosphorylation of RIPK1 (*3*, *4*). Mice and humans with LoF or loss-of-expression (LoE) TBK1 mutations display signs of systemic inflammation due to dysregulated TNF and RIPK1 signaling, which in mice can be partially compensated for by IKKε (*3*, *5*, *7*). Mice with myeloid-specific TBK1 and IKKε deletion have RIPK1-dependent systemic inflammation but intriguingly also show a contribution of IL-1 family cytokines as triple deletion of IL-1R1, IL-18R1, and IL-33R1 strongly reduced systemic inflammation and emergency myelopoiesis (*5*, *45*). We found here that in a macrophage infection model with the RIPK1-activating pathogen, *Y. pseudotuberculosis*, the cell death is RIPK1 driven, yet TBK1/IKKε inhibition also sensitizes cells to enhance secondary NLRP3-dependent caspase-1 activation. The enhanced NLRP3 activation is mediated likely by a combination of a lower threshold of NLRP3 activation, enhanced RIPK1-Caspase-8 signaling, and enhanced alarmin release due to the RIPK1-mediated cell death. This suggests that in the context of TBK1/IKKε inhibition, NLRP3 activation is sensitized to generate mature forms of IL-1 family cytokines, which were elevated in our study and also reported in mice and humans lacking TBK1/IKKε. It further implies that during episodes of infection, in people with TBK1 mutations, the NLRP3 pathway may be hyperactivated in macrophages, following either direct activation, as we have observed with *Listeria*, or downstream of RIPK1, as seen following *Yersinia* infection, and thus will greatly contribute to immunopathology. We show that acute exposure of TBK1/IKKε-inhibited macrophages to TNF, in the presence of a pathogenic NLRP3 activation signal such as nigericin, induces a strong and rapid NLRP3-dependent cell death response, which at later time points displays both RIPK1 and NLRP3 pathway activation. These data indicate that TBK1 and IKKε LoF predisposes myeloid cells to both NLRP3- and RIPK1-dependent programmed cell death and that both pathways can be activated in vivo and in vitro to contribute to the overall cell death and inflammation phenotype. Our results and previous research thus prompt the hypothesis that patients suffering from genetic diseases caused by LoF or LoE TBK1 mutations, such as amyloid lateral sclerosis (ALS) or frontotemporal dementia (*46*–*48*), may benefit from dual RIPK1 and NLRP3 inhibitor therapy to ameliorate neural and systemic inflammation, particularly during bacterial infection. While cell death and ALS disease onset in mice are initiated by RIPK1 (*3*), NLRP3 further contributes to disease progression and inflammation (*49*–*51*), strengthening the potential benefit for dual therapy. Conversely, TBK1 inhibition has recently been shown to improve responses to PD-L1 blockade in cancer immunotherapy by lowering the cell death threshold of tumor cells (*52*). TBK1 inhibitors have also been suggested as treatment options for type I interferon–driven chronic inflammatory diseases including those STING-driven (*53*). Whether the chronic, systemic administration of TBK1 inhibitors to treat these pathologies will sensitize some immune cells to premature death and inflammation remains to be investigated.

How TBK1 and IKKε prevent NLRP3-dependent pyroptosis and cytokine secretion remains poorly understood; thus, we aimed to address this question in the present study. We used the acute 30-min inflammasome activation assays to uncouple the previously unknown role of TBK1/IKKε in cell death control from the known role of TBK1/IKKε in LPS-induced transcription. In experiments using actinomycin, monensin, and direct coculture of WT and TBK1/IKKε KO cells, we show that TBK1/IKKε control over cell death is entirely independent of de novo transcription, induced protein secretion or other external secreted or cell-cell contact signals, or any autocrine signaling. TBK1 and IKKε induce glycolysis (*2*, *54*); thus, we tested whether cell death responses might be different because of extracellular influences of different pH levels (*55*) or metabolites (*56*, *57*) acting on NLRP3. SN transfer, ASC speck staining in cocultures of WT and TBK1/IKKε-deficient iBMDMs, protein secretion blockade, and inhibition of new transcription experiments showed no influence by any secreted factors on enhanced NLRP3-driven death of cells lacking TBK1/IKKε. Our results suggested a cell-intrinsic, rapid mode of action of TBK1/IKKε in control of the NLRP3 pathway. We previously described that TBK1/IKKε control the NLRP3 pathway upstream of ASC oligomerization (*6*), but ASC itself was not affected by TBK1/IKKε. In agreement, activation of other ASC-dependent inflammasomes was unaffected by the loss of TBK1/IKKε, ASC protein levels did not change after TBK1/IKKε inhibition (*6*), and only ASC speck formation was increased as a result of enhanced NLRP3 signaling.

In our previous work, one site of TBK1/IKKε action was AKT, a proposed kinase for the NLRP3 inhibitory phosphorylation on serine 3. However, we found that expression of an unphosphorylated NLRP3 serine 3 mutant (S3A) did not eliminate the TBK1/IKKε control over the NLRP3 pathway, suggesting a second place of TBK1/IKKε control in the NLRP3 pathway. To map the place of TBK1/IKKε action in the NLRP3 pathway, we found here that they control NLRP3 downstream of potassium efflux, upon nigericin stimulation. NLRP3 activation, upstream of ASC speck formation and downstream of potassium efflux, requires NLRP3 trafficking to negatively charged PI4P-positive vesicles, a step regulated by IKKβ (*38*, *39*). Accumulating evidence identified NLRP3 recruitment to these vesicles as a crucial requirement for NLRP3 activation (*17*–*19*) downstream of all NLRP3 activating stimuli. As TBK1/IKKε are known regulators of IKKβ activity and also known regulators of vesicle trafficking in cells, we investigated the influence of TBK1/IKKε inhibition on endocytic vesicle homeostasis and NLRP3 recruitment to TGN46-positive vesicles. To our surprise, TBK1/IKKε inhibition resulted in disruption of the compact TGN and partial dispersion of the TGN46^+^ vesicle network, creating a primed platform for NLRP3 activation. This was not sufficient to recruit NLRP3 to the vesicles and still required signal 2 (nigericin) likely to expose the negatively charged PI4P on the TGN46-positive vesicles (*18*) and/or to induce potassium efflux for NLRP3 conformational changes (*58*). We also found that disrupting endosomal homeostasis by knocking down the early endosomal effector protein Rab5 recapitulated the phenotype seen upon TBK1/IKKε inhibition and resulted in strongly enhanced NLRP3 activation and cell death in response to signal 2 alone. These findings are in line with the previously described role of Rab5 in limiting NLRP3 activation (*59*) and suggest that any perturbation of endosomal homeostasis, either through loss of TBK1/IKKε or disruption of Rab5, signals cell stress and lowers the threshold for NLRP3 activation. Whether TBK1/IKKε and Rab5 form a part of the same signaling axis is impossible to test without knowing whether there is a phospho-residue on Rab5 directly regulated by TBK1, which remains to be tested. NLRP3 is typically activated on Rab5^+^EEA1^+^ vesicles (*18*). In agreement with our findings in macrophages, previous work in neurons (*44*) also reported that TBK1 deletion results in the accumulation of early Rab5^+^EEA1^+^ vesicles and the complete loss of downstream Rab7 vesicles. The only currently known target of TBK1 in the Rab axis is Rab7, by direct phosphorylation of at S72 (*42*, *43*) . We have shown that in macrophages as well, Rab7 phosphorylation is lost in cells lacking TBK1/IKKε activity. However, Rab7 deletion by siRNA-mediated knockdown did not recapitulate the phenotype of cell lacking TBK1/IKKε, suggesting that, as proposed in neurons, in macrophages as well, the loss of TBK1/IKKε signaling arrests endosomal homeostasis upstream of Rab7 vesicles. It will be interesting to test in the future how TBK1 and IKKε regulate early endosomal homeostasis and whether Rab5 vesicles are directly controlled by TBK1/IKKε in the NLRP3 pathway (*59*, *60*).

In conclusion, our study shows that TBK1 and IKKε regulate both NLRP3- and RIPK1-driven cell death and shows that their deletion or inhibition in myeloid cells results in excessive activation of both death pathways. Further, it shows that TBK1/IKKε are important for maintaining endosomal homeostasis, and, if their signaling is disrupted, the resulting accumulated vesicles lower the threshold for NLRP3 activation and permit premature pyroptosis.

## MATERIALS AND METHODS

### Mice and cells

As we previously described, C57BL/6 mice were purchased from Charles River. In-house bred C57BL/6 mice were used as a source of bone marrow cells for the generation of BMDMs in vitro. All mice were housed and bred under specific pathogen–free conditions, and all studies were performed following the ethical standards approved by the Home Office and the University of Oxford (*6*). WT, *Tbk1*^−/−^, *Ikk*ε^−/−^, and *Tbk1*^−/−^*Ikk*ε^−/−^ (DKO) iBMDMs were generated by D.D.N.’s laboratory, as described previously (*1*). *Tnfr1*^−/−^ bone marrow and WT controls were a gift from R. Williams. *Atg7*^−/−^ bone marrow and WT controls were a gift from K. Simon. *Ripk1*^D138N/D138N^ bone marrow and WT controls were a gift from P. Broz.

### Generation of primary mouse BMDMs

BMDMs were generated as previously described by differentiating them from mouse bone marrow for 6 days with recombinant M-CSF (macrophage colony-stimulating factor, 50 ng/ml, BioLegend, 574808) (*6*). In brief, cells were cultured in complete macrophage medium consisting of DMEM (Dulbecco’s modified Eagle’s medium, Gibco, 41965039) with 10% FBS (fetal bovine serum, Gibco, certified low endotoxin, 1600044), 1× penicillin-streptomycin/glutamine (Gibco, 10378016), and 20 mM Hepes [2-(4-(2-hydroxyethyl)-1-piperazinyl)-ethanesulfonic acid, Gibco, 15630056] at 37°C with 5% CO_2_ and supplemented with 5 ml of fresh media containing M-CSF (50 ng/ml) on day 3. After day 6 of differentiation, the cells were counted and frozen down. For experiments, the cells were thawed and rested for 1 day in a complete medium containing M-CSF (50 ng/ml) before plating and stimulated the next day corresponding to day 8 of differentiation.

### Generation of HMDMs

Peripheral blood mononuclear cells were isolated following previously described procedures described in (*6*) using Ficoll gradient from healthy donors from NHS Oxford blood bank (REC 11/H0711/7). CD14^+^ magnetic beads (eBioscience, 8802-6834-74) were used to positively select monocytes. CD14^+^ cells were differentiated into macrophages by culturing them for 7 days with M-CSF (100 ng/ml, BioLegend, 574808). Cells were cultured in RPMI 1640 (Gibco, 21870076) supplemented with 10% FBS (Gibco, certified low endotoxin, 1600044) and 1× penicillin-streptomycin/glutamine (Gibco, 10378016) at 37°C with 5% CO_2_ and supplemented with fresh media containing M-CSF (100 ng/ml) on day 2 and day 5. After day 7 of differentiation, the cells were replated, and on day 8, the cells were stimulated for inflammasome experiments.

### Inflammasome stimulation

Differentiated primary mouse BMDMs were plated at a density of 1 × 10^6^ cells/ml in DMEM supplemented with M-CSF (50 ng/ml) and immortalized BMDMs were plated at 0.75 to 1 × 10^6^ cells/ml in DMEM. Differentiated HMDMs were plated at a density of 0.7 × 10^6^ cells/ml in RPMI 1640 media supplemented with M-CSF (100 ng/ml).

For NLRP3 activation, the cells were primed for 0.5 to 4 hours with *Escherichia Coli* K12 ultrapure LPS (100 ng/ml; Invivogen, tlrl-peklps) for primary BMDMs and HMDMs or LPS (1 μg/ml) for iBMDMs. The cells were subsequently stimulated with 5 to 7.5 μM or 10 to 15 μM nigericin (Sigma-Aldrich, N7143-5MG) for primary BMDMs/HMDMs and iBMDMs/HEK293T-NLRP3 FLAG cells, respectively, for 1 hour or as indicated in figure legends. Potassium efflux–independent NLRP3 activation was induced by the treatment of primed BMDMs with 70 μM R837 (Invivogen, tlrl-imq) for 1.5 hours. For ATP and MSU stimulations, primary BMDMs were primed with LPS (100 ng/ml) for 0.5 hours, followed by stimulation with 5 to 10 mM ATP or MSU crystals (500 μg/ml) for 1.5 or 4 hours, respectively. For AIM2, inflammasome activation was induced by transfecting iBMDMs with calf-thymus DNA (100 ng per well) with 0.25% Lipofectamine 2000 and incubation for 4 hours, and NLRC4 activation was induced by iBMDMs transfection with flagellin (50 ng per well) and 0.25% FuGene HD. For NLRC4 and AIM2 stimulation, the cells were spun for 10 min at 1000*g* after adding stimulants to synchronize transfections.

### Inhibitors and chemicals

The following molecule inhibitors and chemicals were used at the following concentrations unless indicated otherwise in figure legends: MRT68601 (1 to 10 μM, Tocris Bioscience, 5067), MRT67307 (1 to 10 μM, Tocris Bioscience, 5134), MCC950 (10 μM, Merck, 5.38120), 7-Cl-O-Nec1 (50 μM, Abcam, ab221984), actinomycin D (5 μg/ml, Sigma-Aldrich, A1410), Q-VD-Pph (10 μM, Selleck Chemicals, S7311), Z-VAD-FMK (10 to 20 μM, Selleck Chemicals), monensin (2 μM, Invitrogen, 00-4505-51), BI605906 (10 μM, Tocris Bioscience, 5300), brefeldin (5 μg/ml, BioLegend, 420601), bafilomycin A1 (10 nM, Sigma-Aldrich, B1793), Smac mimetic AZD 5582 (0.5 to 2 μM, Tocris Bioscience, 5141), 0.5 μM TAK1 inhibitor 5z-7-oxozeaenol (MedChem Express, HY-12686), and P2X7 inhibitor (10 μM, Tocris Bioscience, 3323).

### Cell death assays

#### LDH release

LDH release was used to measure cell death using the Cytox96 nonradioactive cytotoxicity assay (Promega, G1780) as previously described (*6*). In brief, equal amounts of cell-free SNs from stimulated cells were mixed with the LDH reagent and incubated until the colorimetric signal from the lysis control had fully developed. Reactions were stopped by the addition of an equal amount of stop solution (Promega), and absorbances at 490 and 690 nm were measured on a FLUOstar Omega microplate reader. The percentage of LDH release was calculated relative to 100% cell lysis in untreated control samples lysed with 0.1% Triton X-100.

#### PI uptake

PI (Sigma-Aldrich, 4864) uptake was used to measure cell permeabilization. Cell stimulations were carried out in 90 μl of Opti-MEM in 1% FBS, and 10 μl of Opti-MEM supplemented with PI was added at the time of addition of the inflammasome activation signal with a final concentration of 1 μg/ml of PI. PI signals were analyzed every 30 to 60 min by measuring fluorescence on a FLUOstar Omega microplate reader at λ_excitation_ = 544 nm and λ_emission_ = 620/10 nm. The percentage of PI uptake was calculated relative to 100% control lysis cells.

### K^+^-efflux inhibition

To test the influence of potassium efflux on NLRP3 activation, 5 × 10^4^ BMDMs were plated the day before the experiment. The next day, the medium was removed and replaced with Opti-MEM supplemented with 1% FBS. The cells were stimulated with inhibitors and the addition of 50 mM potassium chloride (KCl) for 30 min before stimulation with 7.5 to 10 μM nigericin followed by kinetic measurements of PI uptake.

### SN transfer

For SN transfer experiments, WT or *Tbk1*^−/−^*Ikk*ε^−/−^ iBMDMs were plated at 0.75 × 10^6^ cells/ml in an F-bottom 96-well plate the day before the experiment. The next day, the medium was aspirated and replaced with fresh cDMEM in which the cells were incubated for 30 min. Then, conditioned media were collected and spun in a U-bottom plate for 3 min and 1500 rpm to obtain a cell-free SN. The medium of a second plate of WT and *Tbk1*^−/−^*Ikk*ε^−/−^ iBMDMs was aspirated and replaced with the conditioned medium. NLRP3 inflammasome activation was then induced by priming cells with LPS (1 μg/ml) for 30 min and stimulation with 15 μM nigericin followed by harvest of the SNs and cell lysis for further analysis.

### Cytokine analysis

As previously described (*6*), secretion of IL-1β, IL-18, and TNF-α were monitored in cell-free SNs using enzyme-linked immunosorbent assay (eBioscience, 88-7013-77, 88-7324-77, and BMS618-3TEN). IL-1β cleavage and release were measured in cell SNs and cell lysates by immunoblot using anti–IL-1β antibody (R&D Systems, AF-401-NA) to measure pro–IL-1β as well as the cleaved p17 fragment.

### Immunoblotting

For immunoblotting analysis, the cells were lysed in denaturing SDS lysis buffer [2% SDS and 66 mM tris (pH 7.4)]. We separate SNs from the remaining cell extracts (XTs) upon cell death, so we could use the same SN for immunoblot, LDH release cell death assay, and cytokine secretion assay. As cells undergo lytic death, they will lose some of the GAPDH from the XT and it will appear in the SN. We show one example of “whole-well lysis” (containing SN + XT) compared to separately run SN and XTs (fig. S1D) to help understand the biochemical analyses shown in the rest of this study. Samples of denatured SN and XT were prepared as described previously (*6*), by mixing with 4× Laemmli buffer and dithiothreitol at a final concentration of 20 mM and boiled at 95°C for 5 min. The samples were then run on 4 to 20% SDS–polyacrylamide gel electrophoresis gradient gels to separate proteins and transferred to nitrocellulose membranes via semidry transfer (Trans-Blot Turbo, Bio-Rad). Following the transfer, the membranes were blocked in 5% nonfat milk or 5% bovine serum albumin (BSA) in tris-buffered saline (TBS) [50 mM tris/HCL and 150 mM NaCl (pH 7.6)] supplemented with 0.1% Tween-20 (TBS-T) for 1 hour at room temperature. Primary antibodies were added in 2.5% NFM (nonfat milk) or 2.5% BSA in TBS-T supplemented with 0.02% sodium azide overnight at 4°C. Membranes were washed the next day and incubated with horseradish peroxidase–conjugated secondary antibodies at a 1:5000 dilution in 2.5% NFM in TBS-T for 1 hour at room temperature. Unbound secondary antibodies were removed by washing with TBS-T before exposing membranes to chemiluminescence detection reagents (ECL or ECL-plus, Bio-Rad). The membranes were developed using x-ray films on an Agfa Curix 60 developing machine. Antibodies used were as follows: GAPDH (1:5000, Cell Signaling Technology, 2118), tubulin (1:1000, Sigma-Aldrich, T5168), caspase-1 (1:1000, Adipogen, AG-20B-0042-C100), IL-1β (1:1000, R&D Systems, AF-401), ATG7 (1:1000, Cell Signaling Technology, 8558), TBK1 (1:1000, Cell Signaling Technology, 3504), IKKε (1:1000, Cell Signaling Technology, 3416), IKKβ (1:1000, Cell Signaling Technology, 8943), phospho-IKKα/β (1:500, Cell Signaling Technology, 2694), caspase-8 (1:1000, Cell Signaling Technology, 9746), cleaved caspase-8 (1:1000, Cell Signaling Technology, 9429), LC-3 (1:1000, Sigma-Aldrich, L8918), RIPK1 (1:1000, Cell Signaling Technology, 3493), and RIPK3 (1:1000, Cell Signaling Technology, 95702).

### *L. monocytogenes* infection

For infection of iBMDMs with *L.m*. 0.75 × 10^6^ WT or *Tbk1*^−/−^*Ikk*ε^−/−^ iBMDMs were plated in antibiotic-free DMEM in an Flat-bottom 96-well plate. At the same time, an overnight culture of *L.m.* [described in (*61*)] was started by picking one colony of *L.m*., grown for a day on LB agar from frozen log stock, and transferring to 5 ml of antibiotic-free LB medium. The next day (16 hours after starting the bacterial culture), the medium was aspirated from the iBMDMs and replaced with fresh cDMEM containing 10 μM MCC950 or a vehicle control and cells were incubated for 30 min. During inhibitor incubation, the bacteria were harvested by centrifugation (10 min, 3000 rpm) and resuspended in an equal volume of phosphate-buffered saline (PBS). The medium from iBMDMs was aspirated and replaced with different dilutions of *L.m.* in PBS, or PBS only as a nonstimulated control. The lowest-dose MOI for this acute assay was 30. MCC950 or vehicle was replenished to avoid wash-out. The cells were then spun for 5 min, 1500 rpm to synchronize the infection and incubated for 1 hour before cell death analysis by LDH and cell lysis for immunoblotting.

### MOI determination for *L. monocytogenes* infection

The MOI for *L. monocytogenes* infections was determined with overnight cultures of *L.m.* used for infection experiments. In brief, one colony of *L.m*., grown for a day on LB agar from frozen log stock, was transferred to 5 ml of antibiotic-free LB medium. The next day (16 hours after starting the bacterial culture), bacteria were spun down and resuspended in the same amount of fresh LB medium following the iBMDM infection protocol with PBS. Multiple log dilutions of the bacterial cultures were spread on antibiotic-free LB agar plates and incubated for 24 hours at 37°C. The next day, the plate with the highest amount of separable colonies was counted and used for MOI calculations.

### *Y. pseudotuberculosis* infection

Primary BMDMs were seeded a day before infection at in 96-well plate at 5 × 10^4^ cells per well in antibiotic-free DMEM supplemented with 10% FBS, 20% L929 media (as a source of M-CSF), nonessential amino acid, GlutaMax, and Hepes. On the day of infection, DMEM was replaced with fresh Opti-MEM. In parallel, overnight *Y. pseudotuberculosis* [described in (*62*)] was diluted in fresh 2× YT media supplemented with 20 mM magnesium chloride and 20 mM sodium oxalate and grown for 1 hour at 26°C followed by another 2 hours at 37°C with aeration. Log phase bacteria were washed thrice with warm Opti-MEM and used to infect BMDMs at a MOI of 5 and 7.5. The plates were centrifuged at 300*g* for 5 min at 37°C to synchronize infection, and gentimicin (100 μg/ml) was added at 1 hour post-infection to kill extracellular bacteria. Wherever indicated, BMDMs were treated with 50 μM Nec-1s or 10 μM MRT67307 or 10 μM of the P2X7 inhibitor AZ10606120 for 30 min before *Y. pseudotuberculosis* infection. For SN transfer, WT or Ninjurin-1 lysis-deficient K45Q-mutant BMDMs (this mouse is generated by K. Chen and will be described in a separate study) were infected with *Yptb* at MOI 7.5 for 3 hours and cell viability was measured by LDH. SNs were collected, treated with a high concentration gentamicin (200 μg/ml), and transferred onto naïve BMDMs pretreated for 30 min with 10 μM MRT67307. After 3.5 hours, caspase-1 and caspase-8 activation were assessed in recipient cells by immunoblot.

### Lentiviral transduction to generate HEK293T-NLRP3-FLAG cells

Lentiviral particles were produced by transfecting 4 × 10^6^ HEK293T cells with 0.75 pmol psPAX2, 0.75 pmol pMD2.G, and 1.5 pmol of the NLRP3-FLAG expression vector (pLV backbone, VectorBuilder). In brief, one tube containing the vectors and 21.3 μl of P3000 reagent (Invitrogen, L3000015) in Opti-MEM was mixed with a second tube containing 25 μl of Lipofectamine-3000 in Opti-MEM. Transfection mixes were incubated for 20 min at room temperature and added drop-wise to HEK293T cells. The medium was changed 6 to 8 hours posttransfection to cDMEM. Viral SNs were harvested 48 hours posttransfection, filtered through a 0.45-μm low-protein binding filter, and mixed with polybrene (Merck, TR-1003-G) to a final concentration of 6 μg/ml. For HEK293T transduction, 1 × 10^5^ HEK293T cells per well were plated the day before in six-well plates. The next day, the medium was aspirated and replaced with 2 ml of the viral SN-polybrene mix. Plates were spun at 1000*g* for 1.5 hours at 32°C and then topped up with 2 ml of cDMEM. Cells were incubated overnight, and the medium was changed on day 2 post-posttransduction to 2 ml of cDMEM supplemented puromycin (1 μg/ml) to begin selection until nontransduced control cells died.

### Immunostaining and fluorescence microscopy

HMDMs (5 × 10^4^ per well) were plated in eight-well NUNC chamber slides, or 8 × 10^4^ NLRP3-FLAG HEK293T cells per well were plated in poly-l-lysine–coated Permanox chamber slides. The cells were stimulated the next day with 3 μM MRT68601 for 30 min, followed by stimulation with 7.5 μM or 2.5 to 10 μM nigericin for HMDMs and HEK293T, respectively. HMDMs were treated with 10 μM of the caspase-1 inhibitor VX-765 at the time of MRT68601 treatment to prevent lytic cell death. The cells were then washed once with PBS and fixed with 4% paraformaldehyde (PFA) for 30 min at room temperature. All stainings were carried out at room temperature in a humidified chamber under light exclusion. The cells were permeabilized with 0.1% Triton X-100 in PBS for 10 min followed by three washes with PBS. The cells were then blocked with 10% FBS in PBS to prevent nonspecific antibody binding for 30 min and then incubated with primary antibodies against TGN46 (Bio-Rad, AHP500G, 1:50 dilution) and/or FLAG (Invitrogen, PA1-984B, 1:1000-1:2000 dilution) diluted in 2% FBS in PBS for 2 hours. The slides were washed three times with PBS + 0.1% Tween-20 (PBS-T) and incubated with secondary antibodies, donkey anti-sheep AF-647 (Invitrogen, A-21448, 1:500), and goat anti-rabbit AF-568 (Invitrogen, A-11011; 1:1000) for 1 hour in 2% FBS in PBS. The cells were washed in PBS-T and mounted with ProLong Diamond mountant containing 4′,6-diamidino-2-phenylindole. The cells were imaged as Z-stacks on a Zeiss LSR980 confocal microscope. Z-stacks were collapsed using Fiji for visualization and analysis.

### ASC speck microscopy in iBMDM coculture

To assess a contribution of soluble factors on ASC speck formation in TBK1/IKKε-depleted cells, a coculture of 1 × 10^5^ cells from each WT and TBK1^−/−^/IKKε^−/−^ DKO iBMDMs was plated in the same well in an eight-well microscopy slide and incubated overnight. The next day, the cells were primed with LPS (1 μg/ml) in the presence of the caspase-1 inhibitor VX-765 to prevent lytic cell death. NLRP3 was activated by stimulation of cells with 15 μM nigericin for multiple time points (1, 2, and 3.5 hours) after which the cells were washed with PBS and fixed with 4% PFA for 0.5 hours. The cells were then subjected to the immunostaining protocol with an anti-ASC antibody (Adipogen, AG-25B-0006-C100, 1:250 dilution). The cells were imaged on a Zeiss 980 confocal microscope. WT and DKO iBMDMs were differentiated on the basis of GFP signal in DKO iBMDMs (*1*). The percentage of ASC^+^ cells was determined by counting all GFP^−^ and GFP^+^ cells and ASC specks from multiple fields of views from three independent experiments to calculate the ratio of ASC^+^ cells for each genotype.

### Statistical analysis

Unless stated otherwise, in vitro data were analyzed using Prism Software V10 and two-way analysis of variance (ANOVA) with multiple-column comparisons using Prism Software V10 and one-way ANOVA with multiple-column comparisons. In all experiments, *P < 0.05, **P < 0.01, ***P < 0.001, ****P < 0.0001.
