# Supplementary material for: TBK1 and IKKε prevent premature cell death by limiting the activity of both RIPK1 and NLRP3 death pathways
Source: Sci Adv. 2025 Mar 7;11(10):eadq1047. doi: 10.1126/sciadv.adq1047 (PMC11887814; doi:10.1126/sciadv.adq1047)
Supplement: Supplementary file 1 — Figs. S1 to S8 [file sciadv.adq1047_sm.pdf]

Science Advances
AAAS

Supplementary Materials for

TBK1 and IKKε prevent premature cell death by limiting the activity of both RIPK1 and NLRP3 death pathways

Fabian A. Fischer *et al.*

Corresponding author: Jelena S. Bezbradica, jelena.bezbradica@kennedy.ox.ac.uk

Sci. Adv. **11**, eadq1047 (2025)
DOI: 10.1126/sciadv.adq1047

This PDF file includes:

Figs. S1 to S8

Supplemental Figure 1

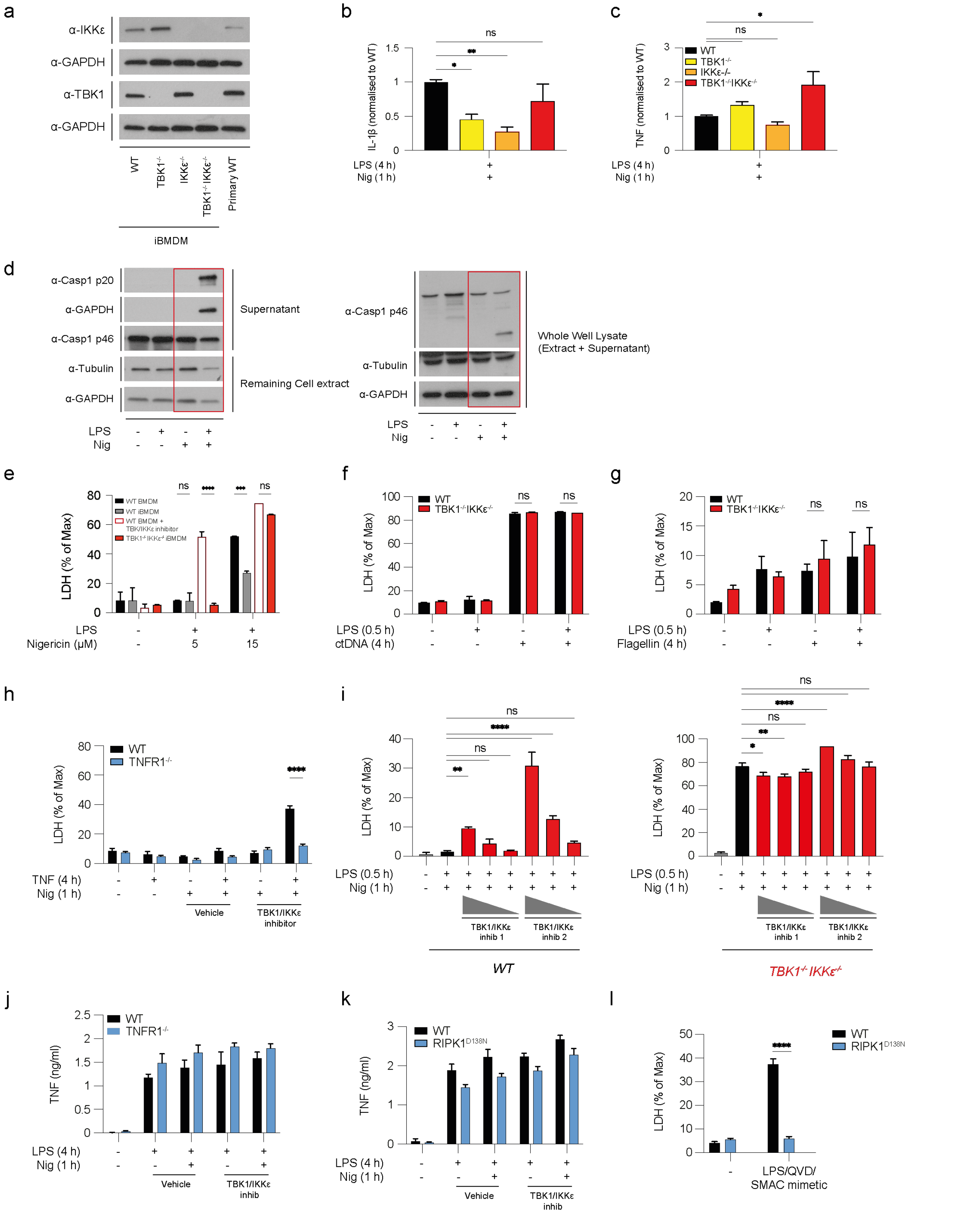

Supplementary Fig.1 | TBK1/IKKε are involved in the transcriptional priming downstream of TLRs, hence the use of transcription-independent acute assays in this study. Control simulations confirm the expected phenotypes of *Tnfr1*$^{-/-}$ and *Ripk1*$^{D138N/D138N}$ BMDMs.

(a)TBK1 and IKKε expression were monitored by Immunoblot in WT, TBK1 KO (*Tbk1*$^{-/-}$), IKKε KO (*Ikkε*$^{-/-}$), TBK1 (*Tbk1*$^{-/-}$*Ikkε*$^{-/-}$) iBMDMs or primary BMDMs (b,c) WT, TBK1 KO (*Tbk1*$^{-/-}$), IKKε KO (*Ikkε*$^{-/-}$) or TBK1 (*Tbk1*$^{-/-}$*Ikkε*$^{-/-}$) iBMDMs were primed with 1 µg/ml LPS for 4 h followed by stimulation with 10-15 µM nigericin for 1 h. (d) The supernatant (SN) and cell extracts (XT) of WT BMDMs were analyzed separately by immunoblot or pooled together into a whole well lysate. (f, g) WT and *Tbk1*$^{-/-}$*Ikkε*$^{-/-}$ iBMDMs were primed with 1 µg/ml LPS for 30 min and stimulated with calf-thymus DNA (ctDNA) or flagellin for AIM2 and NLRC4 inflammasome activation respectively for 4 h. (h) WT and *Tbk1*$^{-/-}$*Ikkε*$^{-/-}$ iBMDMs were primed with 1 µg/ml LPS for 30 min in the presence of dose titrations of the TBK1 inhibitors MRT67307 (inhibitor 1) or MRT68601 (inhibitor 2) ranging from 1-10 µM and then stimulated with 10-15 µM nigericin for 1 h. (i, j, k) WT, *Tnfr1*$^{-/-}$ or *Ripk1*$^{D138N/D138N}$ BMDMs were primed with 100 ng/ml LPS or 100 ng/ml TNF for 4 h followed by stimulation with 7.5 µM nigericin. Cells were treated 30 min before nigericin with 3 µM of the TBK1/IKKε inhibitor MRT68601. (l) WT or *Ripk1*^{D138N} BMDMs were stimulated with 100 ng/ml LPS, 10 µM of the pan-Caspase inhibitor QVD and 0.5 µM of the SMAC mimetic AZD5582. (e, f, g, h, I, l) Cell viability was measured using LDH release. (b, c j, k) Cytokine release was measured using ELISA. Data are shown as mean + SD of duplicates or triplicates from four independent experiments for (b, c), three independent experiments for (f) two independent experiments for (e, g, h, i, j, k, l) and one representative immunoblot shown (a, d).

Supplemental Figure 2

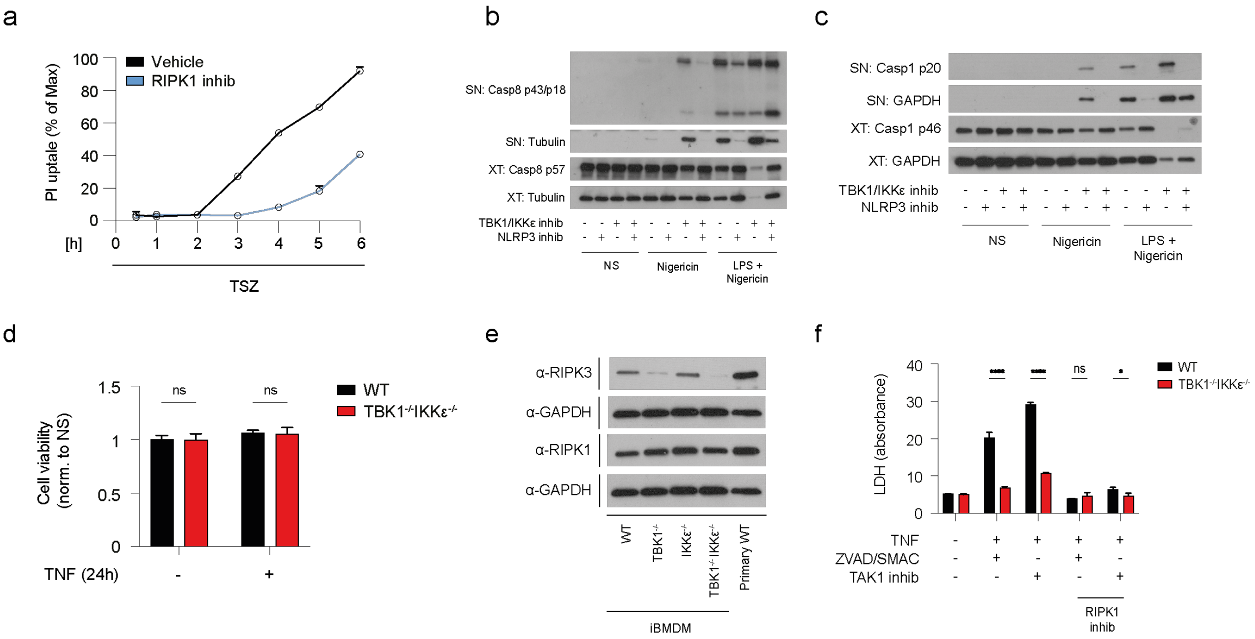

Supplementary Fig.2 | Caspase-1 and -8 are activated in macrophages upon TBK1/IKKε inhibition.
(a) Necroptosis in WT BMDMs was activated by stimulation with 100 ng/ml TNF, 0.5 µM SMAC mimetic AZD5582 and 10 µM pan-Caspase-1 inhibitor Z-VAD (TSZ) in the presence of 50 µM of the RIPK1 inhibitor Nec-1. Cell viability was measured using PI uptake (b, c) Caspase-8 and Caspase-1 cleavage were assessed in cell extracts (XT) and supernatants (SN) from Fig.2 after 6 h by immunoblot. Data are shown as mean + SD from one experiment for (a) and representative immunoblots in (b, c). (d) WT and *Tbk1*$^{-/-}$Ikkε$^{-/-}$ iBMDMs were stimulated with 100 ng/ml TNF for 24 h cell death was measured using CellTiterGlo and cell viability was normalized to unstimulated controls. (e) Relative expression of RIPK1 and RIPK3 in resting WT, *Tbk1*$^{-/}$, *Ikkε*$^{-/}$-, *Tbk1*$^{-/-}$*Ikkε*$^{-/-}$ iBMDMs or primary WT BMDMs were measured by immunoblot. (f) WT and *Tbk1*$^{-/-}$Ikkε$^{-/-}$ iBMDMs were stimulated with 100 ng/ml TNF with or without 0.5 µM TAK1 inhibitor or 2 µM ZVAD/SMAC mimetic for 4 h. Cell death was measured using LDH release. Data are shown as mean + SD of triplicates from three independent (d, f) or one (a) experiments and representative immunoblots for (b, c) from two independent experiments.

Supplemental Figure 3

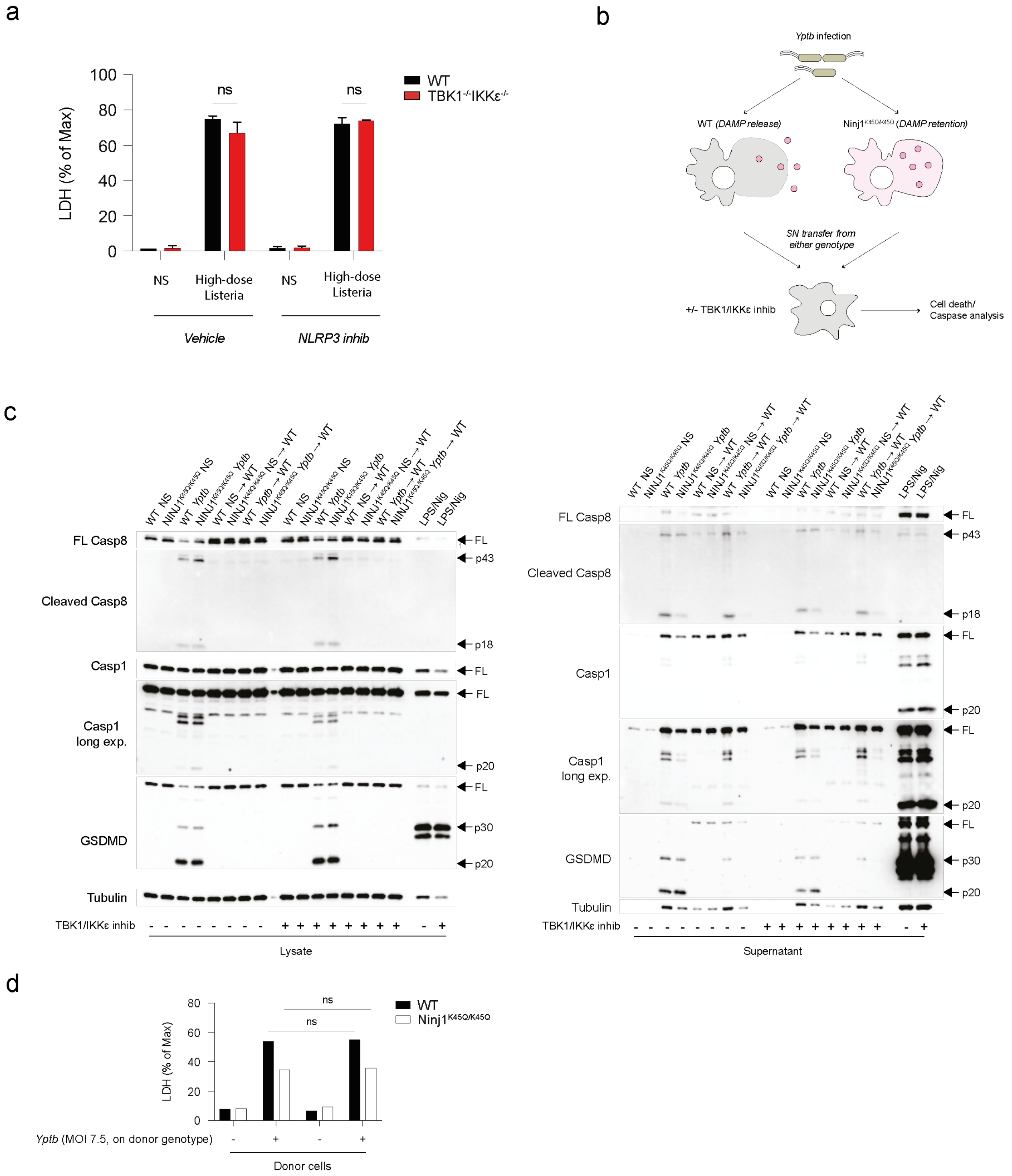

Supplementary Fig.3 | *Listeria* infection dose titration; and *Yersinia* infection supernatant transfer set-up.
(a) WT or *Tbk1-/-Ikkε-/-* iBMDMs were pretreated with 10 μM MCC950 (NLRP3 inhib) for 30 min and then stimulated with high-dose listeria (pure listeria from overnight culture reconstituted in PBS) for 1 h. (a) Cell

viability was measured using LDH release. Data are shown as mean + SD of duplicates from one representative experiment of two independent repeats. (b, c, d) For supernatant transfers, WT or NINJ1 lysis-deficient mutant BMDMs were infected with *Yptb* at MOI 7.5 for 1 h, supernatants were collected, treated with antibiotics and transferred onto naïve BMDMs pre-treated for 30 min with 3 μM MRT68601. After 1h, Caspase-1 and Caspase-8 activation were assessed in recipient cells by immunoblot and cell viability of donor cells was measured using LDH. Data are shown as representative immunoblots of two independent experiments.

Supplemental Figure 4

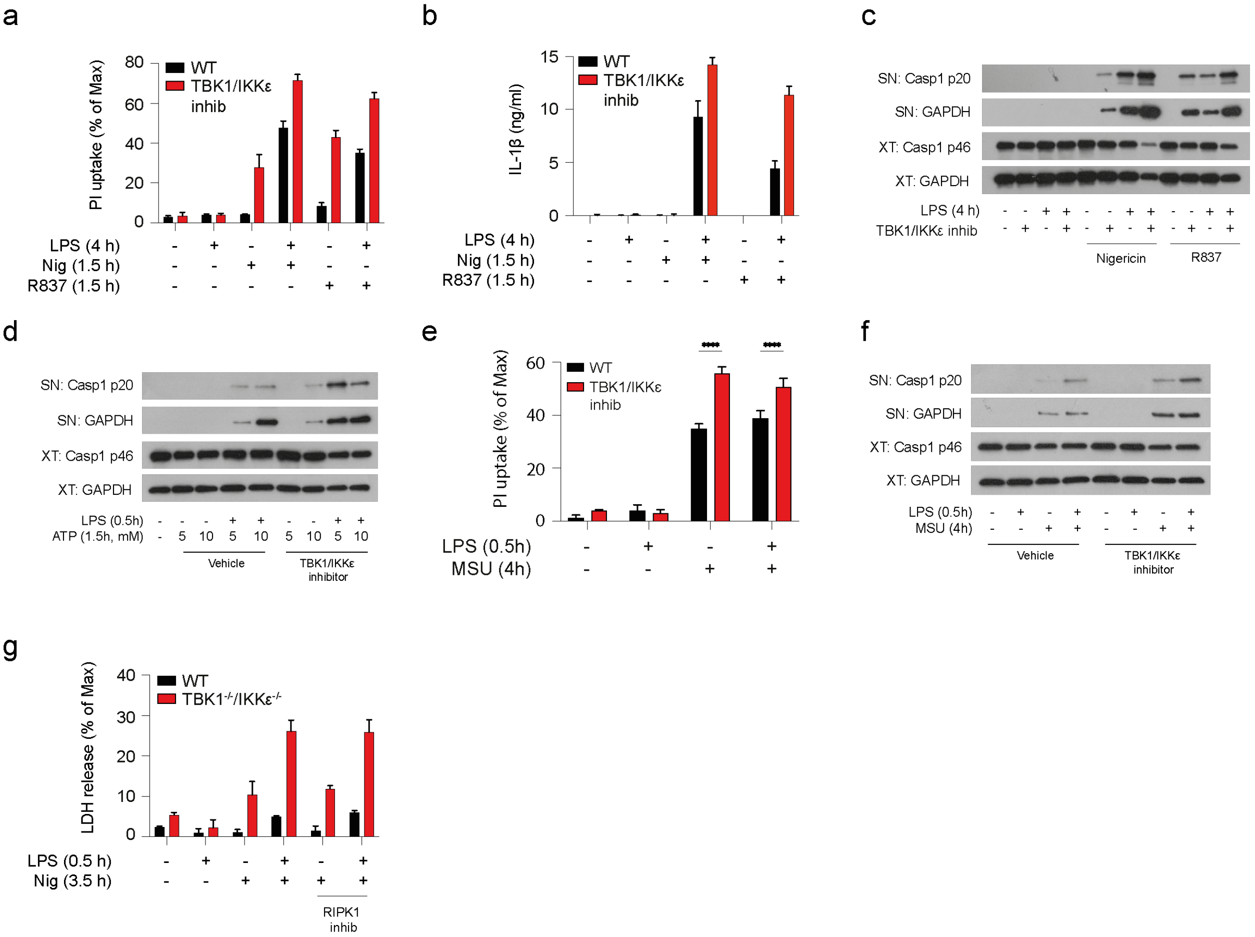

Supplementary Fig.4 | TBK1/IKKε increase NLRP3 activity and death in response to potassium efflux-dependent and -independent activation signals; responses are RIPK1-independent.

(a-f) BMDMs were primed with 100 ng/ml LPS for 0.5 h or 4 h followed by stimulation with various signal 2 activators of NLRP3: 7.5 µM nigericin, 70 µM R837 for 1.5 h, titrating mM doses of ATP for 1.5h, or 500 µg/ml MSU for 4 h. Cells were treated with 3 µM MRT68 for 30 min before signal 2 addition (a-c) or at the time of LPS priming (d-f). (g) WT or *Tbk1*$^{-/-}$*Ikkε*$^{-/-}$ iBMDMs were primed with 1 µg/ml LPS for 1 h followed by stimulation with 15 µM nigericin for 3.5 h. Cell viability was measured using LDH release (g) or PI uptake (a, e). (b) Cytokine release was measured using ELISA. (c, d, f) Caspase-1 cleavage was measured in supernatants (SN) and cell extracts (XT) using immunoblotting. Data are shown as means + SD of triplicates (a, b, e) or duplicates (g) from three (a, b, d, e, f) or two (g) independent experiments and one representative blot is shown for (c, d, f).

Supplemental Figure 5

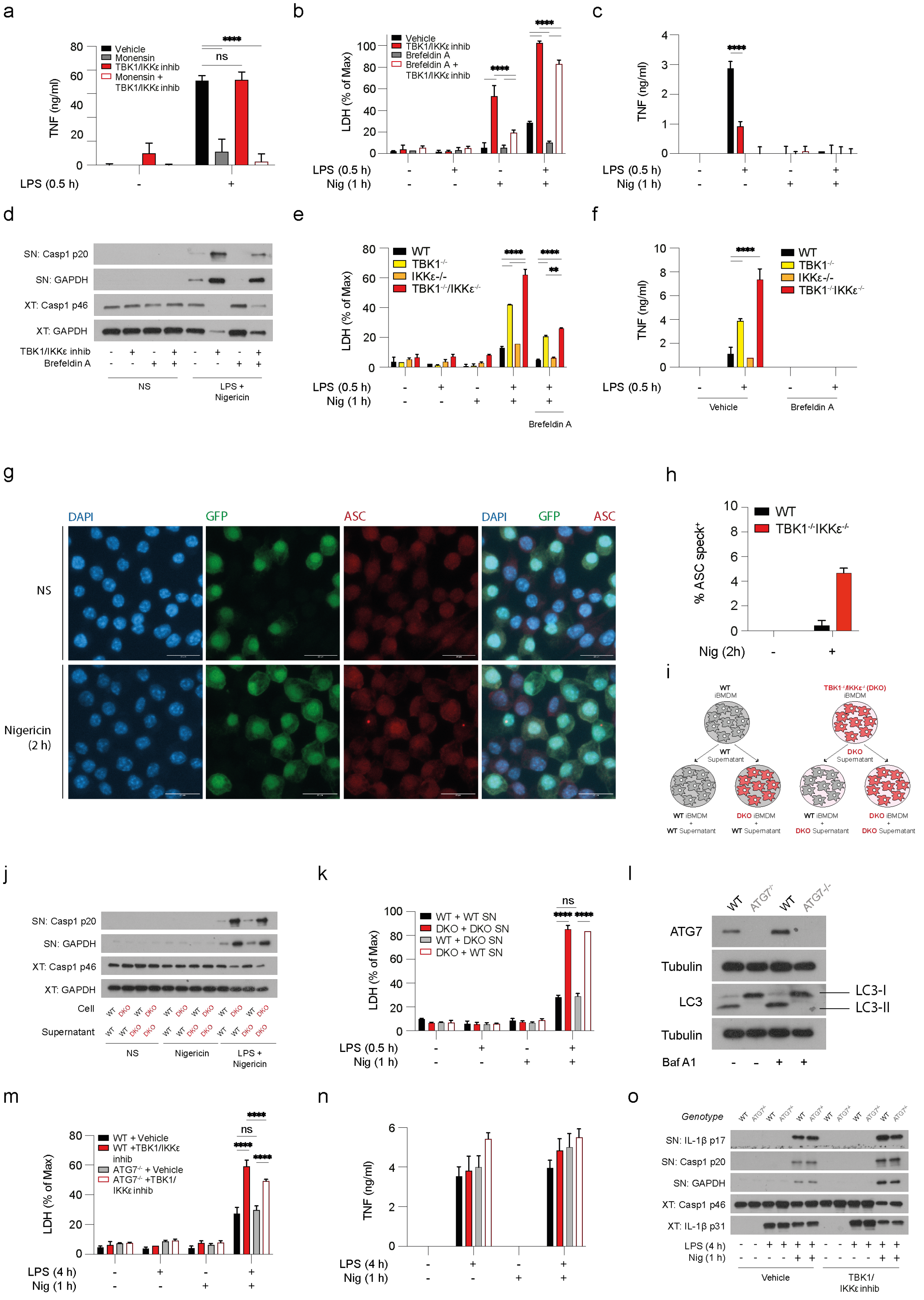

Supplementary Fig. 5 | TBK1/IKKε regulate NLRP3 activation by a cell-intrinsic mechanism, independent of new transcription or autophagy.

(a, b, c, d, m, n, o) BMDMs were primed with 100 ng/ml LPS for 30 min or 4 h followed by stimulation with 7.5 μM nigericin for 1 h. Cells were pretreated with 3 μM MRT68601 (TBK1/IKKε inhib) for 30 min before LPS treatment and 2 μM Monensin or 5 μg/ml. Brefeldin A for 45 min before LPS treatment (e, f) WT, *Tbk1*$^{-/-}$, *Ikkε*$^{-/-}$ or *Tbk1*$^{-/-}$*Ikkε*$^{-/-}$ iBMDMs were primed with 1 μg/ml LPS for 30 min followed by stimulation with 10-15 μM nigericin for 1 h. (g,h) WT or *Tbk1*$^{-/-}$*Ikkε*$^{-/-}$ iBMDMs were cocultured in the same dish for 24 h before being stimulated with 15 μM nigericin for 2 h, without LPS priming, in the presence of 50 μM of the Caspase-1 inhibitor 50 μM VX-765 to prevent lytic cell death. ASC specks were visualized by confocal imaging using an anti-ASC antibody and DAPI stain or WT (GFP^{-}) and *Tbk1*$^{-/-}$*Ikkε*$^{-/-}$ (GFP^{+}) iBMDMs. Data are shown as collapsed Z-stacks of representative images and quantification showing % cells forming ASC specks from multiple fields of view from two independent experiments. (i, j, k) Cultured supernatants were exchanged between WT or *Tbk1*$^{-/-}$*Ikkε*$^{-/-}$ iBMDMs before they were primed with 1 μg/ml LPS for 30 min followed by stimulation with 10-15 μM nigericin for 1 h. (l) WT or Atg7$^{-/-}$ BMDMs were stimulated with 10 nM Bafilomycin A1 for 2 h before immunoblot analysis. (b, e, k, m) Cell viability was measured using LDH release. (a, c, f, n) Cytokine release was measured using ELISA. (d, i, o) Caspase-1 processing was measured in supernatants (SN) and cell extracts (XT) using immunoblotting. (l) Genotypes and autophagy induction were measured using immunoblotting. Data are shown as mean + SD of triplicates (a, b, c, m, n) or duplicates (e, f, k) from one representative experiment of two (a, b, c, e, f, m, n) or four (k) independent experiments and one representative immunoblot in (d, i, l, o).

Supplemental Figure 6

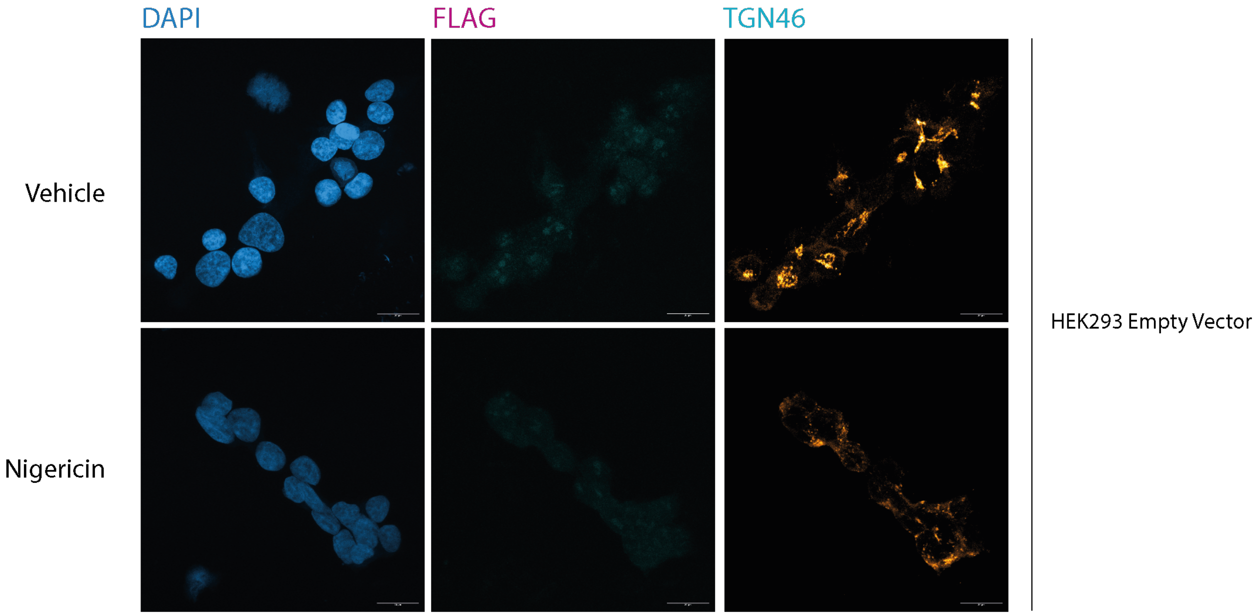

Supplementary Fig. ***6*** **| Lack of NLRP3-FLAG staining at TGN46+ vesicles in control cells not expressing NLRP3-FLAG.**

Control HEK293T cells not expressing NLRP3-FLAG were stimulated with 10 µM nigericin for 1 h. Cells were then fixed and immuno-labelled for TGN46 and FLAG.

Supplemental Figure 7

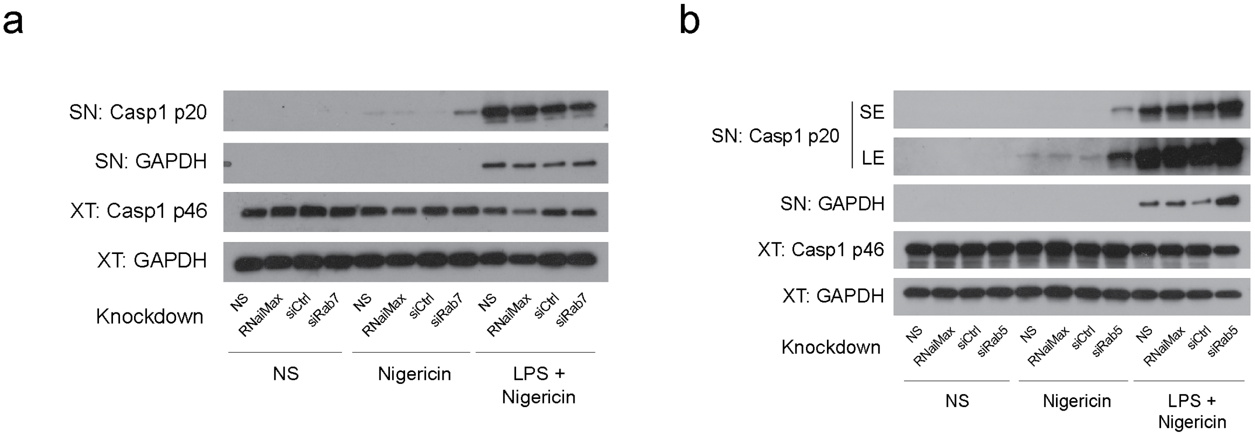

Supplementary Fig. 7 | Deletion of endosomal regulator Rab5 recapitulates phenotype seen in TBK1/IKKε–deleted cells.

(a, b) BMDMs from Fig.7 subjected to siRNA-mediated knockdown of Rab7 (a) or Rab5 (b) were analysed after 2 h for Caspase-1 processing in supernatants (SN) and cell extracts (XT). Data are shown as one representative immunoblot.

Supplemental Figure 8

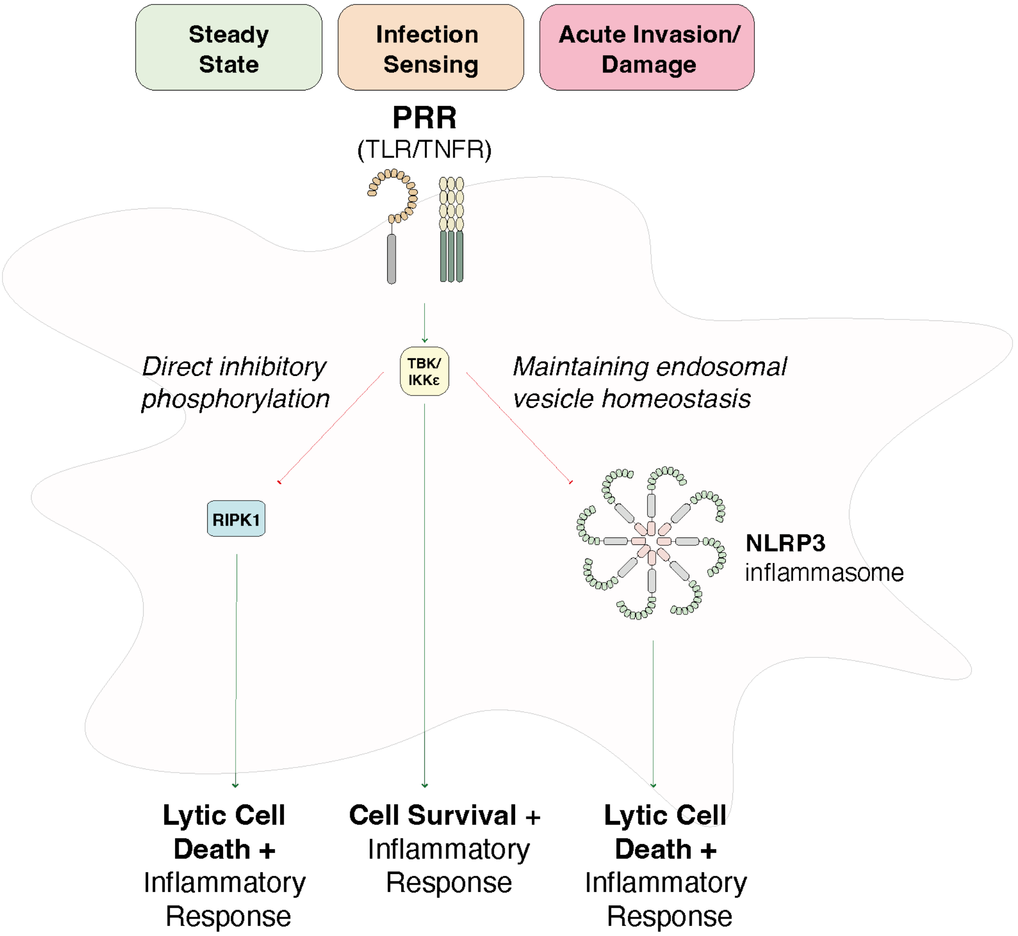

Supplementary Fig. 8 | Model of how TBK1/IKKε prevent cell death: by inhibiting premature activation of RIPK1 and NLRP3 in death-inducing pathways. TBK1/IKKε are activated downstream of Pattern Recognition Receptors (PRR) resulting in the activation of inflammatory responses to allow cell survival and return to homeostasis. Simultaneously TBK1/IKKε also prevent premature death of activated cells, by inhibiting RIPK1-mediated death (via direct inhibitory phosphorylation), and NLRP3-dependent pyroptosis (via maintenance of endosomal homeostasis). Pharmacological inhibition or genetic deletion of TBK1/IKKε predisposes cells to spontaneous TNFR-RIPK1-driven death and also lowers the threshold for NLRP3 activation in response to acute damage or infection signals.
